# Recent Advances in Clustered Regularly Interspaced Short Palindromic Repeats/CRISPR-Associated Proteins System-Based Biosensors

**DOI:** 10.3390/bios15030155

**Published:** 2025-03-02

**Authors:** Xianglin Xin, Jing Su, Haoran Cui, Lihua Wang, Shiping Song

**Affiliations:** 1 Institute of Materiobiology, College of Sciences, Shanghai University, Shanghai 200444, China; xxl23@shu.edu.cn (X.X.); haoran@shu.edu.cn (H.C.); wanglihua@shu.edu.cn (L.W.); 2 School of Perfume and Aroma Technology, Shanghai Institute of Technology, No. 100 Haiquan Road, Shanghai 201418, China

**Keywords:** CRISPR/Cas system, biosensors, biosensing systems, diagnostics

## Abstract

High-sensitivity and high-specificity biodetection is critical for advancing applications in life sciences, biosafety, food safety, and environmental monitoring. CRISPR/Cas systems have emerged as transformative tools in biosensing due to their unparalleled specificity, programmability, and unique enzymatic activities. They exhibit two key cleavage behaviors: precise ON-target cleavage guided by specific protospacers, which ensures accurate target recognition, and bystander cleavage activity triggered upon target binding, which enables robust signal amplification. These properties make CRISPR/Cas systems highly versatile for designing biosensors for ultra-sensitive detection. This review comprehensively explores recent advancements in CRISPR/Cas system-based biosensors, highlighting their impact on improving biosensing performance. We discuss the integration of CRISPR/Cas systems with diverse signal readout mechanisms, including electrochemical, fluorescent, colorimetric, surface-enhanced Raman scattering (SERS), and so on. Additionally, we examine the development of integrated biosensing systems, such as microfluidic devices and portable biosensors, which leverage CRISPR/Cas technology for point-of-care testing (POCT) and high-throughput analysis. Furthermore, we identify unresolved challenges, aiming to inspire innovative solutions and accelerate the translation of these technologies into practical applications for diagnostics, food, and environment safety.

## 1. Introduction

Biosensors are analytical detection systems that convert biological recognition events into useful information. Typical biosensors primarily consist of a biomolecular recognition unit that generates a selective response, a transducer unit that converts the biological recognition information into a digital detection signal, and an output unit responsible for signal amplification and display [1]. Biosensors are widely used in various fields such as life and health sciences, food safety, and environmental protection. They have made significant contributions to detecting disease biomarkers, foodborne pathogens, and monitoring environmental contaminants like antibiotics and heavy metal residues [2,3,4,5,6].

In many practical application fields (clinical diagnostics, scientific research, environmental monitoring, food safety testing, etc.), especially for detecting low-concentration target molecules and complex samples, the specificity and sensitivity of detection are crucial. These factors heavily influence the accuracy and effectiveness of subsequent treatment. High sensitivity and specificity in detection also reduce the possible results of false positives and negatives. In order to enable early intervention in adverse conditions such as diseases and pollution, there is an increasing demand for faster sensing methods. Therefore, new technical means must be employed for developing novel biosensors to meet the growing demand to overcome existing limitations (insufficient sensitivity, lack of specificity, high costs, etc.).

The CRISPR/Cas system plays a significant role in the immune processes of bacteria and archaea. Since it has been discovered in the 1980s, its unique properties (such as specificity, programmability, and accessibility) have attracted extensive attention [7,8,9]. In 2012, Emmanuelle and Jennifer first utilized it as a novel tool for gene editing [10]. Their contributions to this field were recognized with the Nobel Prize in Chemistry award in 2020 [11]. The specific recognition ability of the CRISPR/Cas system for target nucleic acids and its cleavage activity also show great potential in biosensing. It has been successfully extended beyond nucleic acid detection to encompass other non-nucleic acid targets through innovative engineering approaches that integrate intermediate recognition elements and sophisticated signal transduction mechanisms. Owing to their good selectivity and sensitivity [12], CRISPR/Cas system-based biosensors have played important roles in medical diagnoses [13,14,15,16] and environmental and healthy monitoring [17,18,19,20], etc.

This review introduces the different kinds of CRISPR/Cas system-based biosensors developed, discussing their research progress and working mechanisms. This review not only emphasizes the significant advancements in CRISPR/Cas system-based biosensors mostly in the last five years but also provides a systematic classification and analysis based on signal readout modes and structural integration forms. Furthermore, our review focuses on less-explored CRISPR/Cas system-based biosensors and their integration with emerging technologies that are shaping the future of biosensing. This review will primarily focus on the technical applications of CRISPR/Cas system-based biosensors, and therefore, the introduction to the fundamental aspects of CRISPR/Cas systems will be relatively concise. We will also discuss specific limitations and technical challenges associated with these technologies, aiming to provide valuable insights to assist future developments in this field. A comprehensive list of abbreviations is provided following the conclusion section for easy reference.

## 2. CRISPR/Cas System

**Figure 1:**
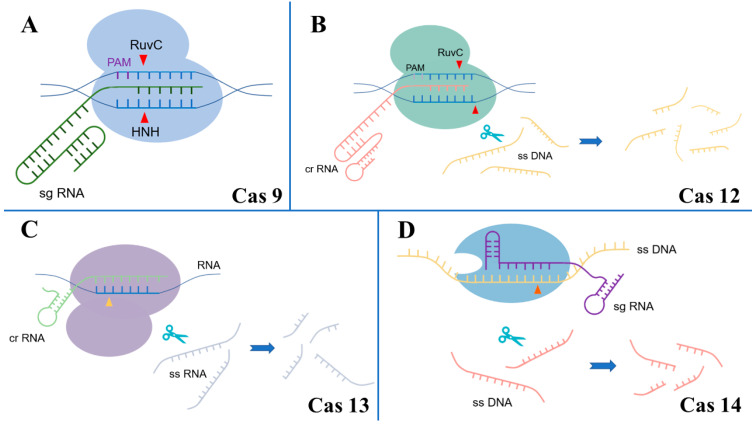
Diagram illustrating four different CRISPR/Cas systems. (**A**) CRISPR/Cas 9; (**B**) CRISPR/Cas 12; (**C**) CRISPR/Cas 13; (**D**) CRISPR/Cas 14.

The CRISPR/Cas system mainly consists of CRISPR RNA (crRNA) and proteins. CRISPR, i.e., Clustered Regularly Interspaced Short Palindromic Repeats, is a special nucleic acid sequence found in bacteria and archaea, used to recognize specific nucleic acid sequences [8,9]. CRISPR-associated proteins (Cas) have enzymatic properties and enable the cleaving of nucleic acid sequences [21,22]. According to the updated classification by Kira Makarova et al. in 2020, the CRISPR/Cas system is divided into two Classes, which are further categorized into 6 types and 33 subtypes. Class 1 (including Type I, Type III, and Type IV) works by relying on multiple effector proteins, while Class 2 only has a single effector protein for function and includes Type II, Type V, and Type VI [23]. The CRISPR/Cas systems execute their biological function via a well-defined three-stage process: adaptation (spacer acquisition), expression, and interference (target cleavage) [24]. The targeting specificity, mediated by guide RNA complementarity, forms the molecular foundation for diverse biosensing applications. The presence and recognition of the Protospacer Adjacent Motif (PAM), located adjacent to the target sequence, serve as an essential prerequisite for the CRISPR/Cas system to initiate its nucleic acid cleavage activity. This PAM-dependent recognition mechanism not only ensures the precise targeting of specific DNA sequences but also contributes to the overall specificity and efficiency of CRISPR/Cas system-based biosensors [25]. The unique enzymatic activity of CRISPR/Cas systems, particularly the *trans*-cleavage activity of Cas12, Cas13, and Cas14 proteins, enables signal amplification essential for sensitive detection. *Trans*-cleavage refers to the nonspecific nuclease activity exhibited by Cas proteins following the recognition and binding of target sequences by the CRISPR/Cas system, which subsequently cleaves arbitrary single-stranded DNA or RNA molecules in the surrounding environment [26,27]. The distinct cleavage characteristics, including both *cis-*cleavage (target-specific) and *trans-*cleavage (non-specific collateral) activities, fundamentally determine the biosensing performance and application scope of different CRISPR/Cas systems. CRISPR/Cas systems used for biosensing are primarily among Class 2, including CRISPR/Cas9, CRISPR/Cas12, CRISPR/Cas13, and CRISPR/Cas14, and their schematic representations are shown in Figure 1.

CRISPR/Cas9 belongs to Class 2, Type II. The full-length sgRNA as a functional component forms a stable complex with Cas9 that facilitates sequence-specific DNA recognition between the guide sequence and the target DNA, with the PAM sequence serving as an additional recognition element. Its target is double-stranded DNA (dsDNA). CRISPR/Cas9 uses the HNH structural domain to cleave the DNA strand complementary to crRNA and the RuvC structural domain to cleave the non-complementary DNA strand [28].

CRISPR/Cas12 belongs to Class 2, Type V. It includes various subtypes such as Cas12a (formerly known as Cpf1), Cas12b (formerly known as C2c1), and Cas12f (formerly known as Cas14a1). Cas12 utilizes sgRNA or crRNA for target recognition and recognizes complementary dsDNA through protospacer, while PAM as a functional motif allows for the re-orientation of the nuclease domains. Only when a PAM is available can ON-target cutting happen. Cas12 has a higher specificity for targeting dsDNA than ssDNA. Its *cis-*cleavage activity uses the RuvC domain, and the additional *trans-*cleavage activity can degrade nearby ssDNA indiscriminately [29].

CRISPR/Cas13 belongs to Class 2, Type VI, formerly known as C2c2. Guided by crRNA, its recognition of ssRNA is based mainly on the protospacer flanking site (PFS), rather than PAM sequences, and cleave targets by using two HEPN domains. Additionally, Cas13 also exhibits *trans-*cleavage activity, enabling it to indiscriminately degrade nearby ssRNA [30].

CRISPR/Cas14 belongs to Class 2, Type II, and represents the smallest known member of the CRISPR/Cas family in terms of molecular weight. Guided by sgRNA, Cas14 can recognize and cleave ssDNA independently of the PAM sequence and activate *trans-*cleavage activity to degrade nearby ssDNA indiscriminately. Meanwhile, Cas14 is sensitive to mismatch near the middle of the target, which inhibits its activity [31].

## 3. CRISPR/Cas System-Based Biosensors

CRISPR/Cas system-based biosensors mainly consist of three core components: (1) CRISPR/Cas system for target recognition, (2) signal transduction elements, and (3) readout systems. Hitherto, a diverse range of biosensors have been developed by leveraging the specificity of both *cis-* and *trans-*cleavage activities of different CRISPR/Cas systems. Leveraging the inherent capability of CRISPR/Cas systems to recognize and cleave specific DNA sequences, biosensors can directly detect target DNA molecules. For non-nucleic acid targets, CRISPR/Cas system-based biosensors incorporate auxiliary recognition elements such as aptamers and antibodies to achieve target-specific detection through indirect mechanisms. Cleavage-based detections measure target presence through the degradation of reporter molecules, while non-cleavage-based approaches primarily utilize target recognition events that trigger subsequent signal transduction pathways. These biosensors play important roles in many fields.

### 3.1. CRISPR/Cas System-Based Biosensors with Electrical Output Signals

#### 3.1.1. CRISPR/Cas System-Based Electrochemical Biosensors

Electrochemical sensors offer advantages such as high sensitivity and selectivity, rapid response, low detection limits, and ease of on-site and real-time monitoring [32]; therefore, CRISPR/Cas System-based electrochemical biosensors are among the most common biosensors, utilizing the CRISPR/Cas system. These biosensors convert specific biological information into electrical signals such as current or impedance to reflect the detection results. They can detect not only pathogens like *Escherichia coli* [33], *Salmonella typhimurium* [34], and the SARS-CoV-2 virus [35], which have extractable genetic information, but also substances closely related to human life, such as cardiac troponin I (cTn I), associated with cardiovascular diseases (CVDs) [36], and even non-biological substances like cocaine [37] and Pb^2^⁺ [38].

**Figure 2:**
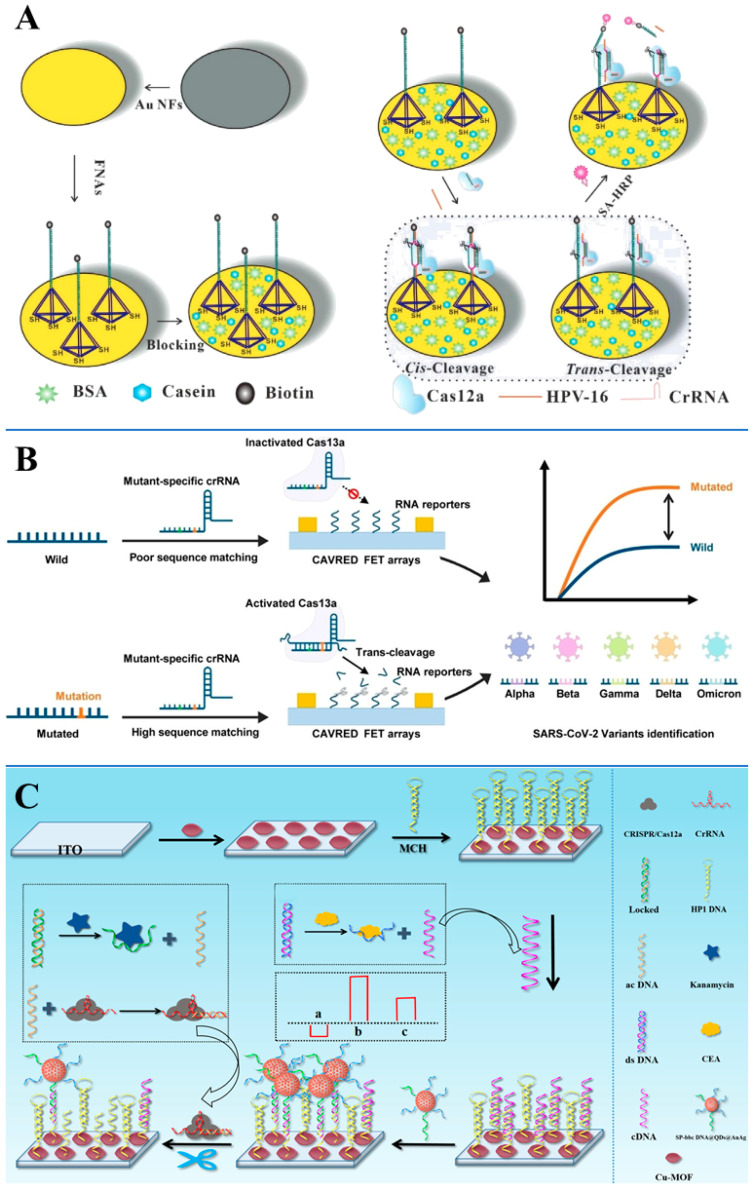
(**A**) Working principle of the FNAs-supported electrochemical biosensing platform in HPV-16 detection. Reproduced with permission from [39]. Copyright 2021, John Wiley and Sons. (**B**) The design of a CRISPR-based Amplification-free Viral RNA Electrical Detection (CAVRED) platform for SARS-CoV-2 and its variants’ detection and identification. Reproduced with permission from [42]. Copyright 2023, John Wiley and Sons. (**C**) Principle of the multifunctional PEC biosensor in CEA and Kana detection (a—no CEA existing; b—CEA existing; c—Kana existing). Reproduced with permission from [43]. Copyright 2023, American Chemical Society.

Our group and Ding’s group co-designed a CRISPR/Cas12a-driven electrochemical biosensor based on DNA framework nucleic acids (FNAs) (Figure 2A) [39]. Gold nanoflowers (AuNFs) electrodeposited at the electrode interface are connected with FNAs through Au-S bonding. The DNA sequence, which is labeled with biotin, complementary to the HPV-16 single-strand target, is the extended sequence of FNAs. It can be used as a reporter probe. When the target HPV-16 sequence is present, Cas12a/crRNA duplexes recognize it, and the crRNA-target forms a complete R-loop with target-reporter DNA, activating the *cis-* and *trans-*cleaving activities of Cas12a. The cleavage reporter probe cannot interact with the streptavidin-horseradish peroxidase (SA-HRP) conjugate that specifically binds on the electrode, and the catalytic reaction that occurs after adding hydrogen peroxide and TMB cannot produce a current response on the electrode. According to the degree of decline of the current signal, a quantitative detection of HPV-16 in the system with a wide dynamic range (100 fM to 100 pM) and a low detection limit (100 fM) is achieved. This biosensor is used with a home-made 16-channel electrochemical workstation, which can analyze multiple samples simultaneously, and it can also detect actual serum samples. This biosensor has good detection stability and universal potential. The uniqueness of this biosensor is that it uses FNAs to improve the accessibility of target and probe molecules while reducing the signal interference of non-specific adsorption on the electrode surface. Meanwhile, by combining the *cis-* and *trans-*cleavage activities of Cas12a, it greatly improves detection sensitivity and simplifies the experimental process effectively.

Xu et al. designed a CRISPR electrochemical biosensor and also utilized this strategy to achieve linear detection of the tumor marker miRNA-19b within a concentration range of 10 pM to 10^4^ pM, with a detection limit of 10 pM [40]. This biosensor, through the interaction of activated Cas13a *trans-*cleavage on the RNA reporter probe modified with avidin-HRP, which was immobilized on the DNA tetrahedral framework structure, and collection of the current signal decay using the electrochemical workstation was performed. Similarly, Cheng et al. developed a CRISPR electrochemical biosensor utilizing a DNA tetrahedron fixed on a gold electrode surface [41]. This approach uses a special DNA tetrahedron that includes unique uracil base sequences. The electrochemically active molecule-methylene blue (MB) is stored within the DNA double helix structure. When the CRISPR/Cas13a system detects the target molecule bladder cancer biomarker circRNA, the activated Cas13a cleaves the uracil base sequences, causing the DNA tetrahedron to break apart. This results in the release of MB from the electrode surface, reducing the electrochemical peak current signal attributed to the redox of MB. The extent of the current signal decrease correlates with the concentration of circRNA in the urine sample.

In addition to being combined with DNA framework structures for electrochemical biosensing, CRISPR/Cas-based electrochemical biosensors can also be paired with hydrogels [44], an electroconductive polymer dot (PD) [45], and other materials for efficient biosensing. Organic framework structures can also achieve effective results in these biosensing applications.

Wang et al. developed an electrochemical biosensor utilizing the ultrathin 2D layered structure of covalent organic framework nanosheets (2D COF NSs) with a high surface area and porosity, loaded with gold nanoparticles (AuNPs) [46]. Based on the CRISPR/Cas12a system, it allows for sensitive detection of a programmed death-ligand 1 protein-positive (PD-L1^+^) exosome with a linear range of 1.2 × 10^2^ to 1.2 × 10^7^ particles/μL and a detection limit of 38 particles/μL. In this biosensor, the AuNPs grown in situ on the COF NSs enhance conductivity. They anchor the DNA activator probes and MB-PD aptamers, which are combined onto the electrode. When PD-L1^+^ is present, the aptamer binds to PD-L1^+^ and releases the activator probe. This interaction triggers crRNA recognition and activates the Cas12a *trans-*cleavage activity, leading to a decrease in the current signal due to the reduced presence of MB molecules on the electrode surface.

Dong et al. combined a metal–organic framework (MOF) with CRISPR/Cas12a to develop an electrochemical biosensor for detecting microRNAs [47]. This biosensor utilizes the porous channels of UiO-66-NH₂ to encapsulate MB and employs ssDNA1 as a biological gate to seal these channels. In the presence of the target miRNA-155, the Cas12a system is activated, and its *trans-*cleavage activity cleaves ssDNA2, which is fully complementary to ssDNA1. As a result, the biological gate is closed, preventing the release of MB from the pores, leading to a weak current signal. Conversely, in the absence of the target molecule, ssDNA2 hybridizes with ssDNA1 to form dsDNA, which reduces the binding affinity, allowing the biological gate to open and produce a significant current signal. The detection limit of miRNA-155 is 31.6 aM.

CRISPR/Cas System-based electrochemical biosensors combine the high specificity of CRISPR/Cas systems with the sensitivity of electrochemical detection, enabling the detection of analytes at ultra-low concentrations (fM to aM range). These biosensors offer rapid, real-time results, making them suitable for point-of-care diagnostics and environmental monitoring. Their wide dynamic range and versatility allow for the detection of diverse targets, including nucleic acids, proteins, and small molecules. Integration with nanomaterials enhances signal amplification and performance, while multiplexing designs increase throughput. Future advancements may focus on expanding analyte detection, improving multiplexing, and integrating these biosensors into portable and wearable devices for real-time monitoring.

#### 3.1.2. CRISPR/Cas System-Based Field-Effect Transistor Biosensors

A field-effect transistor (FET) is a kind of voltage-controlled semiconductor device. A typical FET consists of a pair of source electrodes, drain electrode, and gate electrode. The FET channel connects the source electrode and drain electrode, allowing for current flow. While the voltage between the source–drain electrode remains constant, the magnitude of the current depends on the conductivity of the channel. This conductivity is adjusted by the external electric field provided by the gate electrode, which modulates the carrier density. This method of modulation is known as the field effect [48]. Benefiting from the FET’s high speed, low power consumption, and strong compatibility with integrated circuits, they were widely used in the field of biosensing.

Chen et al. developed a CRISPR-based Amplification-free Viral RNA Electrical Detection platform (CAVRED), utilizing FET biosensors to distinguish between wild and mutant RNA genomes with a low concentration (1 cp/μL) of SARS-CoV-2 detection in a short time and allowing for the differentiation of corresponding variants(Figure 2B) [42]. The *trans-*cleavage function of Cas13a is activated only in the presence of mutant-type virus RNA, leading to the cleavage of RNA reporters anchored to the FET with a negative charge. This results in a change in the surface charge density of the FET, which alters the response current (*I_d_*). By comparing 100 × Δ*I/I_o_* (the percentage of current change(Δ*I*) in before and after target recognition relative to the initial current (*I_o_*) of the FET), the presence of the virus is reflected.

In order to achieve better sensitive detection, graphene-based FET (G-FET/gFET) biosensors, which possess high carrier mobility and good biocompatibility, have come into the limelight. In particular, they have advantages in the sensitive diagnosis of viral infections [49].

Li et al. created a CRISPR-FET biosensor to detect coronavirus (CoV) and hepatitis C virus (HCV) using the CRISPR/Cas13a system and reduced graphene oxide (RGO) FETs modified with AuNPs [50]. The RGO-FET chip is modified with AuNPs, and reRNAs are anchored on the surface of the AuNPs via the Au-S bond to form a multivalent spherical RNA reporter molecule. When the RNA of the target virus is recognized by CRISPR/Cas13a, the *trans-*cleavage activity of Cas13a releases the immobilized RNA reporter molecule, causing a positive shift in the potential of the FET. The size of the shift is correlated with the level of the target virus.

Weng et al. developed a CRISPR Cas12a-gFET biosensor, combining the CRISPR/Cas12a system and gFET arrays [49]. The detection limits of human papillomavirus HPV-16 and *Escherichia coli* are 1 aM and 10 aM, respectively. The ssDNA probes with negative charge are immobilized on the graphene surface, being degraded by *trans-*cleavage-active Cas12a, which is activated in the presence of the target DNA. This leads to a decrease in the gating effect and an increase in the carrier density, resulting in a leftward shift in the charge neutral point (CNP) voltage and enabling the quantification of the target DNA concentration by Δ*V*_CNP_.

CRISPR/Cas system-based field-effect transistor biosensors combine the precise target recognition of CRISPR/Cas systems with the high sensitivity of FETs, enabling the ultra-low concentration detection of analytes such as viral RNA and DNA. These biosensors offer real-time results without the need for target pre-amplification, simplifying workflows and reducing contamination risks. Their integration with nanomaterials, particularly G-FETs, enhances signal amplification and sensitivity due to the high carrier mobility and biocompatibility of graphene. Capable of multiplexed detection, these biosensors are ideal for rapid diagnostics, point-of-care applications, and environmental monitoring.

#### 3.1.3. CRISPR/Cas System-Based Photoelectrochemical Biosensors

The advantages of photoelectrochemical (PEC) biosensors lie in the ultra-high sensitivity, low background signal, sensing mode (focusing on the “signal-on” and “signal-off” modes), and the direction reversal of the photocurrent [51]. This is mainly based on the unique properties of photoactive materials and P-N heterojunctions. As photoelectric transducers, photoactive materials are critical for signal transduction and readout. For example, quantum dots, at a specific quantum size, light absorption efficiency, and the electron transfer rate reach an equilibrium point, possessing the best photoelectrochemical performance. P-type and N-type semiconductors in P-N heterojunctions compete with each other to consume electron donors; therefore, N-type semiconductors can be used as a signal quencher to reduce the cathodic photocurrent of P-type semiconductor-modified electrodes [52].

Li et al. designed a photoelectrochemical biosensor able to detect human papillomavirus HPV-16 in a wide range of 3.0 pM~600 nM with a detection limit of 1.0 pM [53]. The working principle is that the CdS quantum dots and TiO_2_-Au nanorods are connected to the hairpin DNA through the hairpin DNA to form a Z-type heterojunction. When HPV-16 is present in the samples, with the activation of the *trans-*cleavage function of Cas12a protein, the hairpin DNA is damaged, and the Z-type heterojunction is disintegrated; i.e., electrons cannot be transferred through the heterojunction, and the photocurrent is reduced.

Lv et al. developed a photoelectrochemical biosensor that is mainly based on the quenching mode of the P-N heterojunction, and the CRISPR/Cas12a system is utilized to control the switching of this mode to achieve the detection of microRNA (miRNA) [54]. The [(C6)_2_Ir(dcbpy)]^+^PF_6_^−^-sensitized NiO photocathode (P-type semiconductor) has a stable and significant photocurrent signal, which generates an initial PEC signal, and Bi_2_S_3_ quantum dots (N-type semiconductor), which are the signal quenchers, are attached to the photocathode plate by ssDNA to form a P-N heterojunction and are in a bursting state. When the target miRNA is specifically recognized by hairpin DNA, activating the *trans-*cleavage activity of Cas12a, ssDNA is cut off, Bi_2_S_3_ is released and moves away from the P-type semiconductor, and the photocurrent is gradually restored. The detection limit for miRNA is as low as 36 aM, and the linear detection range is 0.1–10 nM.

The quenching and recovery of the photocurrent can be used as signals to indicate the presence or absence of target molecules. Researchers have attempted to investigate whether the flip and increase or decrease in photocurrent can be utilized to detect the target at the same time, and they have realized the detection of two kinds of targets by one biosensor.

Du et al. developed a multifunctional PEC biosensor for the detection of tumor markers-CEA and kanamycin (Kana) using the CRISPR/Cas system, quantum dots (QDs), and MOF (Figure 2C) [43]. Cu-MOF was adsorbed on the electrodes by electrostatic interactions to generate cathodic photocurrents, and hairpin DNA (HP1) was immobilized on the Cu-MOF through amide bonds. The ZnIn_2_S_4_/ZnS QDs, which had particularly strong photoelectric signals, were selected as the signal molecules, and Au-Ag NPs were used as the carriers. They were modified with SP DNA and bbc DNA to form the SP-bbc DNA@QDs@Au-Ag signal probes. When the CEA specifically binds to its aptamer DNA and quantitatively releases cDNAs capable of hybridizing with HP1, the ssDNA formed after HP1-cDNA hybridization becomes partially complementary with the signal probe. It generates a polarity-flipped anodic photocurrent, the intensity of which correlates with the concentration of CEA. When Kana is added, the specific binding of Kana to its aptamer quantitatively releases activator DNA (acDNA), which activates Cas12a *trans-*cleavage activity, and the previously generated anodic photocurrent signal is significantly reduced. The dual detection of CEA and Kana is achieved through this whole process of photocurrent flipping and reduction.

CRISPR/Cas system-based photoelectrochemical biosensors harness the unique properties of photoactive materials and achieve ultra-high sensitivity and low background noise through “signal-on” and “signal-off” modes. These biosensors can detect a broad spectrum of analytes, including nucleic acids, proteins, and small molecules, making them versatile tools for medical diagnostics, environmental monitoring, and food safety. By integrating CRISPR/Cas systems with nanomaterials, signal transduction and amplification are significantly enhanced, enabling the simultaneous detection of multiple targets via photocurrent flipping and reduction. This dual-mode detection capability increases both versatility and efficiency. Compared to traditional methods like ELISA and PCR, CRISPR/Cas system-based PEC biosensors offer faster results, higher sensitivity, and suitability for non-laboratory settings. They also outperform other biosensing technologies, such as optical and electrochemical sensors, by providing lower background noise. Future research may focus on improving the stability and scalability of these biosensors to facilitate their widespread adoption in clinical and environmental applications.

CRISPR/Cas system-based biosensors with an electrical output signal have relatively high sensitivity and involve a wide variety of target analytes. They mainly rely on modification of the electrode interface to improve performance.

### 3.2. CRISPR/Cas System-Based Biosensor with Optical Output Signals

#### 3.2.1. CRISPR/Cas System-Based Fluorescent Biosensors

The fluorescence method, which mainly uses the interaction between fluorescein and a quencher to transform the recognition information of biomolecules, is one of the most convenient biosensing methods, and, therefore, CRISPR/Cas system-based fluorescent biosensors are also one of the most common and widely used types that play an important role in many practical detection scenarios, such as the detection of miRNAs [55], *salmonella* [56,57], African swine fever virus [58], SARS-CoV-2 virus [59,60,61], antibiotics in wastewater [19], etc.

Yu et al. developed a fluorescent biosensor for the detection of organophosphorus pesticides (OPs) in the aquatic environment, utilizing the enzyme catalytic activity of acetylcholinesterase (AChE) and the biorecognition and *trans-*cleavage activity of CRISPR/Cas12a, and the detection limit of dichlorvos (DDVD) in real lake water samples is 0.135 ng mL^−1^ [62]. Activator single-stranded DNA (ssDNA) was bound to manganese dioxide nanosheets by adsorption. The hydrolysis of acetylcholine (ATCh) to thiocholine (TCh) by AChE occurs simultaneously with the reduction of the MnO_2_ nanosheets to Mn^2+^, resulting in the release of the activator DNA, which opens up the *trans-*cleavage function of Cas12a. Then, fluorescent reporter molecule attached to fluorescein FAM and quenchers BQH1 is cleaved, generating fluorescent signals. When the target organophosphorus pesticide is present in the system, it can inhibit the enzyme activity of AChE, silencing the CRISPR/Cas12a system and switching off the fluorescent signals.

Zhou et al. designed a novel fluorescent biosensor, termed HARRY. It also uses typical FAM- and BHQ1-modified ssDNA fluorescent probes (F-Q), which are highly effective in the detection of a wide range of non-nucleic acid target analyses, such as ATP, Cd^2+^, aflatoxin B1 (AFB1), and so on [63]. This biosensor uses CRISPR/Cas14a as the biorecognition and signal initiation unit. Although the recognition between Cas14 and the target DNA does not depend on the PAM sequence, the specificity and sensitivity are very high. The biosensor operates through a unique aptamer–activator (Aptavator) module that exhibits target-dependent regulation of CRISPR/Cas14a activity. It can be specifically recognized by the sgRNA of CRISPR/Cas14a in the absence of the target analyte, forming a stable R-loop structure that activates the *trans-*cleavage function of Cas14a, degrading the nearby F-Q fluorescent probes and generating fluorescent signals. When the Aptavator is bound to the higher-affinity target, the stable conformation reduces its affinity for the complementary nucleic acid, the R-loop in Cas14a is difficult to form, the F-Q fluorescent probe remains stable, and the fluorescent signal disappears.

Both of the above biosensors utilize the typical fluorescence biosensing strategy of “signal on-to-signal off”. For the purpose of avoiding the error caused by fluorescence quench due to changes in external environmental conditions, another fluorescence signal strategy is better.

**Figure 3:**
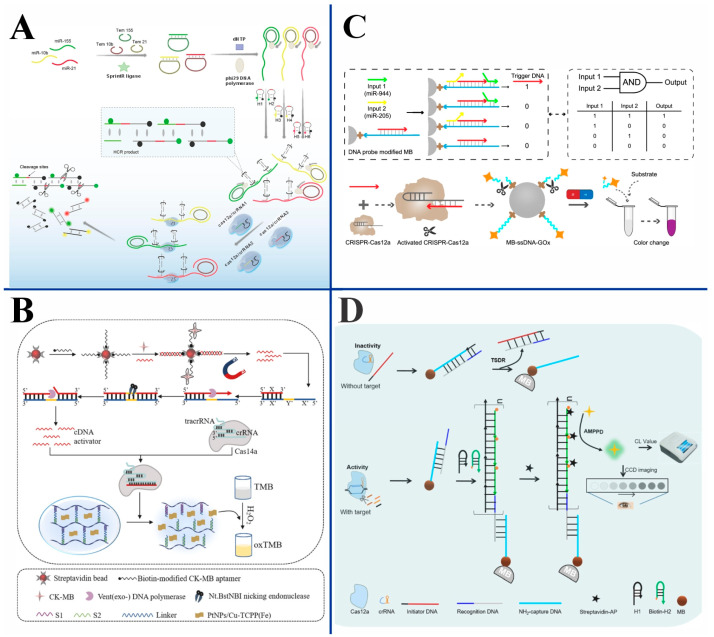
Schematic illustration of (**A**) Cas-Rainbow for the detection of different miRNAs. Reproduced with permission from [64]. Copyright 2024, Elsevier. (**B**) EXPAR-based Cas14a-cleaved DNA hydrogel for creatine kinase isozyme (CK-MB) detection. Reproduced with permission from [65]. Copyright 2022, Elsevier. (**C**) The developed biosensor with AND logic gate and CRISPR/Cas12a system for dual miRNA colorimetric detection. Reproduced with permission from [66]. Copyright 2022, American Chemical Society. (**D**) CLE-CRISPR platform for nucleic acid detection. Reproduced with permission from [67]. Copyright 2022, Elsevier.

Zhu et al. developed a Cas-Rainbow multiplexed fluorescent biosensor with the signal “off-to-on” strategy (Figure 3A) [64]. The use of multiple different fluorescent groups to label reporter gene probes enables the detection of multiple miRNAs, with detection limits of 33 fM, 41 fM, and 79 fM for miR-155, miR-10b, and miR-21 in serum, respectively. The miRNAs were paired with padlock probes (Tem 155, Tem 10b, and Tem 21) to form a SplintR ligase-catalyzed loop DNA template for a rolling circle amplification (RCA) reaction in the presence of dNTP and phi29 DNA polymerase. The obtained RCA product opens the hairpin reporter probe H1 to pair with the complementary H2, triggering the occurrence of the hybridization chain reaction (HCR); at the same time, the RCA product also possesses sequences specifically recognized by CRISPR/Cas12a, which activates the *trans-*cleavage activity of Cas12a, and the three sets of probes (H1 and H2, H3 and H4, H5 and H6) labeled with different fluorescent moieties (Fam, Cy3 and Cy5) formed HCR products with the presence of single-stranded exposed fragments susceptible to degradation by activated Cas12a, resulting in fluorescent signals at different wavelengths.

CRISPR/Cas system-based fluorescent biosensors combine the high specificity of CRISPR/Cas systems with the sensitivity of fluorescence detection, enabling the detection of analytes at ultra-low concentrations. These biosensors are highly versatile, capable of detecting nucleic acids, proteins, small molecules, and environmental contaminants, making them suitable for different applications. They employ both “signal-on” and “signal-off” strategies, allowing for flexible detection mechanisms. The biosensor enables the simultaneous detection of multiple targets using different fluorescent labels, enhancing throughput and efficiency. These biosensors provide real-time results, making them ideal for point-of-care applications. While they compete with electrochemical and other optical biosensors, their ease of use, versatility, and multiplexing capabilities give them a distinct advantage. Future development may focus on visualizing fluorescence signals to enable more convenient and user-friendly point-of-care testing.

#### 3.2.2. CRISPR/Cas System-Based Colorimetric Biosensors

Pursuing simpler and faster readout methods has always been a key focus in biosensing. Colorimetric methods, which determine results by simple spectrophotometric measurements or even by the naked eye, are particularly favored. Therefore, studies on CRISPR/Cas-based colorimetric biosensors are also popular.

Chen et al. designed a colorimetric biosensor based on the CRISPR/Cas14a system, which trigger a TMB colorimetric reaction by degrading DNA hydrogel through the CRISPR/Cas14a system. This biosensor has a detection limit of 0.355 pM (15.62 pg mL^−1^) for creatine kinase-MB (CK-MB), a crucial clinical marker for acute myocardial infarction (AMI), which is far below the clinical abnormal detection range (39–185 ng mL^−1^) (Figure 3B) [65]. Streptavidin-modified magnetic nanoparticles can bind to biotin-modified CK-MB aptamers. The cDNA, which can be specifically recognized by the CRISPR/Cas14a system, competes with CK-MB for binding to the aptamer. When CK-MB is present, the cDNA, which cannot complementarily pair with the aptamer, is captured and recognized by the CRISPR/Cas14a system, activating the *trans-*cleavage activity of Cas14a. This leads to the degradation of the DNA hydrogel, encapsulating platinum nanoparticle-modified metal–organic framework nanosheets (PtNPs/Cu-TCPP(Fe)). The released PtNPs/Cu-TCPP(Fe) catalyze the chromogenic reaction through their peroxidase-like activity, generating a measurable colorimetric signal. The concentration of CK-MB can be quantitatively determined through optical measurement following reaction termination.

In addition to the traditional mode, the CRISPR/Cas system-based colorimetric biosensors can be combined with logic gates to achieve co-detection of multiple targets.

Gong et al. developed a colorimetric biosensor that combines an AND logic gate with the CRISPR/Cas12a system, allowing for the visual detection, with low concentrations (1 pM), of target miRNA through color changes (Figure 3C) [66]. This biosensor can detect the overexpression of miR-205 and miR-944 in the serum of patients with lung cancer, with an instrument detection limit as low as 36.4 fM. Magnetic beads modified with DNA probes are used to recognize the binary input of miRNA. When none or only one of the target miRNAs is present, the AND logic gate outputs a signal of 0. Only when both miRNAs are simultaneously present does the AND logic gate output a signal of 1, releasing the activator DNA. This activator DNA is specifically recognized by the CRISPR-Cas12a system, which activates the *trans-*cleavage activity of Cas12a. This activity cleaves the ssDNA linking the magnetic beads and glucose oxidase (GOx). After magnetic separation, GOx remains in the supernatant, catalyzing a color reaction with the chromogenic substrate. The higher the concentration of the target analytes, the deeper the color of the solution.

The newest smart readout can also be implemented in CRISPR/Cas system-based colorimetric biosensors.

Tao et al. designed a dual-functional DNA probe, utilizing both the CRISPR-Cas12a system and the nanozyme MxeneDNA-Ag/Pt for dual-signal amplification. Combined with smartphone software, this design enables the colorimetric biosensor to achieve real-time detection of the hepatitis B virus (HBV) [68]. DNA metallization can precisely control the growth of metal nanostructures, enhancing the catalytic activity of nanozymes. The specially designed DNA probe serves as both a synthesis template for bimetallic Ag/Pt nanoparticles and an enhancer of Mxene catalytic activity. In the absence of the target substance, the DNA probe precisely regulates the growth of Ag/Pt nanoparticles on Ti_3_C_2_T_x_ MXenes, increasing the peroxidase-like activity of the MxeneDNA-Ag/Pt nanocomposite. When HBV is present, it activates the *trans-*cleavage ability of Cas12a, leading to the degradation of the DNA probe. Although Ti_3_C_2_T_x_ MXenes have some catalytic ability, it is significantly less than that of the MxeneDNA-Ag/Pt nanocomposite. Consequently, the color reaction of TMB (3,3′,5,5′-tetramethylbenzidine) becomes noticeably weaker, and the color of the solution lightens. Using a smartphone to capture the signal, HBV in human serum can be detected quantitatively.

CRISPR/Cas system-based colorimetric biosensors offer a simple and rapid detection method through spectrophotometric measurements or naked-eye observation, making them ideal for point-of-care applications. By combining the high specificity of CRISPR/Cas systems with the sensitivity of colorimetric detection, these biosensors achieve ultra-low detection limits, even below clinically relevant thresholds, enabling early disease diagnosis. They are versatile; capable of detecting proteins, nucleic acids, and other biomarkers; and can be integrated with logic gates for multiplexed target detection or a smartphone for real-time analysis. The use of CRISPR/Cas systems with nanozymes or peroxidase-like materials enables dual-signal amplification, enhancing sensitivity and reliability. Compared to traditional methods, they provide faster results, higher sensitivity, and suitability for non-laboratory settings. Future research may focus on developing more distinguishable color indicators and integrating these biosensors into portable and wearable devices for real-time monitoring.

#### 3.2.3. CRISPR/Cas System-Based Chemiluminescence Biosensors

Chemiluminescence (CL) reactions have high sensitivity and fast response times. Compared to fluorescence signals, CL signals have a lower background and are less prone to quenching, making them an ideal method for biosensing. Particularly, CL signals combined with catalytic coupling systems involving typical enzymes like HRP, alkaline phosphatase (ALP), and magnetic beads (MB) have been successfully applied in commercial diagnostic techniques.

Apart from the most common luminol luminescence mechanism, many new chemiluminescent substrates have also been used in the process of biosensor action.

Zhou et al. designed a CL biosensor for detecting the biomarker microRNA-21 (miRNA-21). It uniquely employs a cation exchange reaction to toggle the signal between “ON” and “OFF” states. CuS nanoparticles (NPs) are immobilized on a microplate via biotin–streptavidin recognition [69]. Ag^+^ ions replace a significant amount of Cu^2+^ through a cation exchange reaction, which then catalyzes the luminol-H_2_O_2_ reaction to produce a strong CL signal. In the presence of the target molecule, the *trans-*cleavage activity of CRISPR/Cas12a is activated, causing most of the Cu^2+^ to be released from the microplate before the exchange occurs. Following the washing steps, the released Cu^2+^ is removed from the reaction system, preventing it from being replaced and used in the subsequent chemiluminescence reaction, thereby reducing the CL signal.

Ke et al. developed a CL-enhanced biosensor that employs a HCR amplification strategy combined with the CRISPR/Cas12a system to detect HPV in clinical samples, achieving a sensitivity of 88.89% and a specificity of 100% (Figure 3D) [67]. In the absence of the target, the initial DNA (intDNA) undergoes a toehold-mediated strand displacement reaction (TSDR) with MB@crDNA, which inhibits further reactions with hairpin DNAs (H1 and H2). When the target DNA is present, the activated *trans-*cleavage activity of CRISPR/Cas12a degrades the intDNA sequence. The ends of MB@crDNA then induce the HCR between H1 and biotin-labeled H2, forming a long, double-stranded DNA framework. This framework adsorbs the streptavidin-AP enzyme, which catalyzes the chemiluminescent substrate AMPPD (1,2-dioxetane derivative) to produce a luminescent signal.

Xu et al. constructed a CL biosensor that is in conjunction with reverse transcription recombinase-aided amplification (RT-RAA) and the CRISPR/Cas13a system [70]. Based on a well-established chemiluminescence mechanism, it achieves a detection limit as low as 19.7 fM for H7N9 avian influenza. Biotin-modified RNA probes are linked to magnetic beads, forming an MB-RNA-ALP complex through specific binding with streptavidin-labeled alkaline phosphatase (ALP-SA). In the presence of the target RNA, the activated *trans-*cleavage activity of Cas13a releases ALP. After magnetic separation, the free ALP in the supernatant catalyzes the chemiluminescent substrate CDP-Star, producing a luminescent signal.

CRISPR/Cas system-based chemiluminescence biosensors utilize the high sensitivity and low background noise of CL reactions, making them ideal for detecting low-abundance analytes with rapid signal generation, crucial for point-of-care applications. These biosensors are highly versatile, particularly for detecting nucleic acids, and are widely used in medical diagnostics, environmental monitoring, and food safety. By integrating CRISPR/Cas systems with signal amplification techniques and catalytic coupling systems, they achieve enhanced sensitivity and reliability. Some designs enable multiplexed detection, improving throughput and efficiency. Compared to electrochemical biosensors, they offer lower background noise and superior multiplexing capabilities. Future research may focus on minimizing external light interference and developing advanced signal amplification technologies to further enhance detection efficiency.

#### 3.2.4. CRISPR/Cas System-Based Electrochemiluminescence Biosensors

Electrochemiluminescence (ECL) is a process where electrochemiluminescent substances undergo an electrochemical reaction on the electrode surface, simultaneously triggering a luminescent reaction. This combines the advantages of both electrochemistry and chemiluminescence, offering high sensitivity, a wide linear range, simple operation, and low background signals. QDs and metal nanoparticles are commonly used to construct ECL biosensors.

A highly sensitive ECL biosensor was developed by Lin et al. for detecting DNA adenine methylation methyltransferase (Dam MTase), achieving a low detection limit of 23.4 mU/mL through a dual-signal amplification process [71]. The logarithm of Dam MTase concentration showed a good linear relationship with ECL signal intensity in the range of 5 to 70 U/mL. The high sensitivity of this biosensor is mainly due to the low background ECL signal. This is achieved by modifying the indium tin oxide (ITO) electrode surface with a Nafion polymer membrane, which creates electrostatic repulsion between the negatively charged signal units and the electrode surface, reducing ECL intensity. Additionally, the signal probe Ru-MOF/ssDNA-Fc consists of a ruthenium-based metal–organic framework (Ru-PEI-L-lys-ZIF-8) linked to ferrocene (Fc) via ssDNA. The aromatic Fc has a low oxidation potential, inhibiting the oxidation of Ru(bpy)_3_^2+^ and thereby suppressing the ECL signal. Fc^+^ further quenches the ECL signal produced by Ru(bpy)_3_^2+^/TPrA through energy transfer (with Ru(bpy)_3_^2+*^ as the energy donor and Fc^+^ as the energy acceptor). In the presence of Dam MTase, hairpin DNA (HP) with methylation recognition sites becomes methylated, activating the specific cleavage activity of the restriction enzyme DPnI. The released ssDNA then acts as an activating strand, initiating the *trans-*cleavage activity of CRISPR/Cas12a. The activated Cas12a separates Fc from Ru-MOF, restoring the suppressed ECL signal and allowing Ru-MOF to approach the ITO electrode more easily. The higher the Dam MTase concentration, the stronger the ECL signal.

There are also some interesting works using Fc quenching probes, but they combine different ECL signal molecules or apply different signal modes.

**Figure 4:**
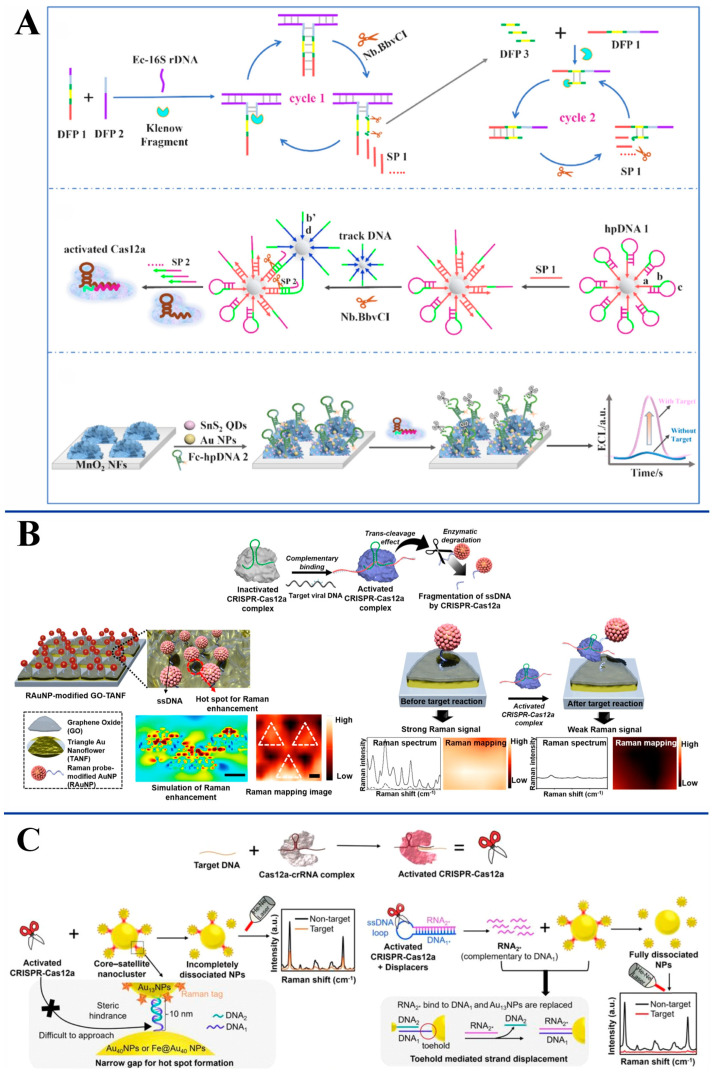
Schematic representation of (**A**) the designed ECL biosensor for *Escherichia coli* detection. Reproduced with permission from [72]. Copyright 2024, Elsevier. (**B**) Surface-enhanced Raman spectroscopy (SERS) biosensor in the sensitive detection of various viral DNA. Reproduced with permission from [74]. Copyright 2021, American Chemical Society. (**C**) CRISPR-Cas12a/SERS integrated biosensing system [75].

Wang et al. used quantum dots to generate ECL signals and used strand displacement amplification (SDA) and dual-particle 3D DNA rollers for cascade signal amplification, designing an ECL biosensor for detecting *Escherichia coli* 16S rDNA (Ec-16S rDNA) (Figure 4A) [72]. This biosensor has a wide response range (100 aM–10 nM) and a low detection limit (27.29 aM). In the presence of Ec-16S rDNA, ssDNA strands DFP 1 and DFP 2 undergo an SDA reaction, producing a large amount of the SP 1 sequence. Hairpin DNA (HP 1) and track DNA are modified on Au NPs. SP 1 opens the stem-loop of HP 1, enabling dual-particle 3D DNA roller replication with track DNA. The introduced Nb endonuclease cuts specific regions of the rollers, generating SP 2 sequences that activate the *trans-*cleavage function of Cas12a. MnO_2_ NFs/SnS_2_ QDs/Au NPs are modified on a glassy carbon electrode (GCE), and Fc is linked to HP 2 via Au-S bonds. Fc presence weakens the ECL signal. When the target gene is present, *trans-*cleavage activity of Cas12a is activated, causing Fc to leave the electrode surface, and the ECL signal generated by SnS_2_ QDs/S_2_O_8_^2−^/MnO_2_ NFs is enhanced significantly. Wei et al. also utilized Fc-modified ssDNA as a quenching probe to develop an ECL biosensor for detecting the SARS-CoV-2 RdRp gene [73]. Through entropy-driven T7 amplification and CRISPR/Cas13a system *trans-*cleavage, the ECL signal switches from “OFF” to “ON”.

Zhang et al. designed another ECL biosensor using N-(4-aminobutyl)-N-ethylisoluminol (ABEI) as the signal molecule [76]. The CRISPR/Cas12a system mediates the on/off state of the ECL signal to detect the cytochrome oxidase subunit I (COI) gene, quantifying the content of adulterated pufferfish meat (because wild pufferfish is toxic and cannot be eaten). NiCo_2_O_4_ NCs@Au-ABEI fixed on the electrode surface generates the ECL signal, while dopamine (DA) is modified on the NiCo_2_O_4_ NCs@Au-ABEI substrate via ssDNA and a tetrahedral DNA scaffold, quenching the ECL signal. When the target gene is present, activated *trans-*cleavage of Cas12a releases DA, restoring the ECL signal and allowing for further quantification of the target molecule through ECL intensity. With the same “OFF-to-ON” signal mode, this biosensor utilizes different ECL signal molecules and special quenching structures to achieve useful applications in food safety testing.

CRISPR/Cas system-based ECL biosensors exploit the high specificity and *trans-*cleavage activity of CRISPR/Cas systems to achieve the ultrasensitive detection of nucleic acid targets, enabling low detection limits through cascade signal amplification strategies. These biosensors are widely applicable in DNA methylation analysis, pathogen identification, and food safety testing. However, challenges such as complex probe design and limited applicability to non-nucleic acid targets hinder their broader use. Future research should focus on integrating CRISPR/Cas systems with aptamers or other recognition elements for non-nucleic acid detection, developing cost-effective and stable variants, enabling multiplexed detection, and combining them with microfluidic platforms for high-throughput, automated analysis.

#### 3.2.5. CRISPR/Cas System-Based Surface-Enhanced Raman Scattering Biosensors

Raman spectroscopy identifies specific chemical structures of biomarkers through the phenomenon of incident light scattering. However, in real samples, the Raman signal intensity of biomolecules at very low concentrations is usually too weak to detect. Surface-enhanced Raman scattering (SERS) enhances the signal through the influence of surface plasmons on metal surfaces, making SERS a highly sensitive and portable tool suitable for the on-site detection of low-concentration biomolecules.

Pan et al., cleverly utilizing Prussian Blue (PB) nanolabels, designed a SERS biosensor to detect adulterants in goat milk [77]. The activated *trans-*cleavage activity of CRISPR/Cas12a releases PB NPs. The remaining PB NPs on the microplate are treated with alkali, producing ferrocyanide anions (Fe(CN)_6_^4−^) that interact with the mixed SERS substrate Au@Ag core–shell NPs in the “biological Raman-silent region”, presenting unique characteristic Raman peaks. Since biological molecules do not typically generate signals in this region, background noise is extremely low, achieving a low detection limit for the target DNA of 224 aM. The Raman intensity difference Δ*I* is linearly related to the target DNA concentration.

Researchers have also made corresponding attempts to analyze whether it is possible to realize the detection of target molecules directly by CRISPR/Cas system-based surface-enhanced Raman scattering biosensors without pre-amplification.

For the sensitive detection of various viral DNAs, Jin-Ha Choi et al. constructed a surface-enhanced Raman spectroscopy (SERS) biosensor without the need for amplification steps, enabling the detection of HBV, human papillomavirus HPV-16, and HPV-18 within a concentration range of 1 aM to 100 pM (Figure 4B) [74]. First, a TANF array composed of periodic triangle Au micropattern and Au nanoflowers structures was constructed. Then, graphene oxide (GO) was modified on its surface. The highly concentrated surface electrons, specially designed micro/nanostructures, and the charge transfer mechanism induced by graphene oxide significantly enhanced the SERS signal on the TANF surface. The surface was also modified with functionalized gold nanoparticle Raman probes (RAuNPs) linked with ssDNA. When the target viral DNA activated the *trans-*cleavage activity of CRISPR/Cas12a, the RAuNPs left the GO-TANF surface, causing a sharp decrease in the SERS signal. The degree of change in the SERS signal reflects the concentration of the target virus.

Yin et al. developed a SERS biosensor, which was based on the CRISPR-Cas12a system for amplification-free nucleic acid detection (Figure 4C) [75]. The ingenuity of the design lies not merely in the activation or deactivation of the *trans-*cleavage activity of Cas12a but in better utilizing SERS through the regulation of the steric hindrance effect and base pairing principle. A core–satellite Au nanocluster structure with ultra-strong SERS signals was constructed as the Raman reporter molecule, connected by partially complementary DNA pairs (DNA_1_ and DNA_2_) linking the surrounding Au_13_ NPs to the central Fe@Au_40_ NPs. When RNA_2*_, which can fully pair with DNA_1_, is released from the hairpin hybridization chain by the activated *trans-*cleavage activity of Cas12a, the toehold-mediated strand displacement reaction (TMSDR) frees the Au_13_ NPs completely from the core–satellite structure. This results in a more significant reduction in the SERS signal, achieving a lower detection limit (10 aM). This design leverages the regulation of the steric hindrance effect and base pairing principle to make the changes in the SERS signals more pronounced and sensitive.

CRISPR/Cas system-based SERS biosensors combine the high specificity of CRISPR/Cas systems, particularly Cas12a, with the ultra-sensitive signal amplification of SERS through plasmonic effects on metal surfaces. These biosensors enable the direct, amplification-free detection of nucleic acids at ultra-low concentrations, leveraging strategies such as the “biological Raman-silent region” and steric hindrance effects to minimize background noise. Despite their high sensitivity and versatility, challenges remain, including the complex and costly fabrication of SERS substrates and susceptibility to environmental factors like temperature and pH. Future efforts should focus on developing cost-effective, scalable substrate fabrication methods and improving signal stability to unlock their full potential in diagnostics and beyond.

For a CRISPR/Cas system-based biosensor with optical output signals, the signal output mode of “off-to-on” is much better than that of light signals from presence to absence, because it can greatly avoid interference from external conditions and prevent the occurrence of false positives, but the latter is still the majority of existing biosensors.

### 3.3. CRISPR/Cas System-Based Biosensors with Other Output Signals

In addition to optical and electrical signals, CRISPR/Cas system-based biosensors can also convert specific biological recognition information into other types of signals, such as magnetic signals and mechanical signals.

**Figure 5:**
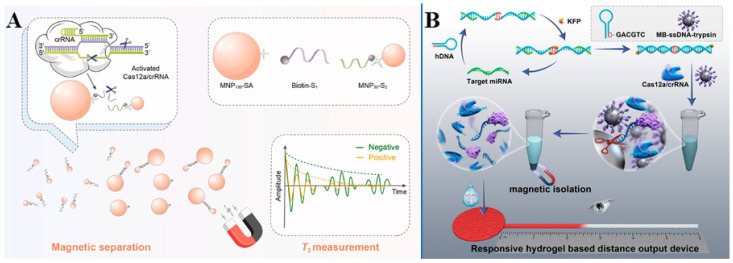
Schematic diagram of (**A**) the CRISPR-MRS biosensor for *Salmonella* detection. Reproduced with permission from [78]. Copyright 2022, Elsevier. (**B**) The designed distance-based biosensor for miRNA detection. Reproduced with permission from [79]. Copyright 2023, American Chemical Society.

Shen et al. designed a biosensing platform to sensitively detect *Salmonella* using the different magnetic properties of magnetic nanoparticles (MNPs) with two sizes (Figure 5A) [78]. Different-sized MNPs primarily function as magnetic separation and signal output agents. To achieve precise control over the binding of the two types of MNPs, the CRISPR-Cas12a system was introduced into this binding process, effectively preventing insufficient crosslinking and nonspecific binding between MNPs and the target. The sensitivity and stability were significantly superior to traditional magnetic resonance sensors (MRS). The MNP_130_-SA and MNP_30_-S_2_ probes, surface-modified with streptavidin (SA) and single-stranded DNA (ss-DNA, biotin-S_2_), respectively, can be connected through complementary biotin-modified ssDNA (biotin-S_1_). When the target *Salmonella* is present, the *trans-*cleavage activity of the CRISPR-Cas12a system is activated, indiscriminately cleaving biotin-S_1_, thereby inhibiting the binding of MNP_130_-SA and MNP_30_-S_2_. The free MNP_30_-S_2_ probes, under an external magnetic field, will not be sedimented and separated like the MNP_130_-MNP_30_ complex and MNP_130_-SA, remaining well dispersed in the solution. The amount of these unbound MNP_30_-S_2_ probes correlates with the concentration of *Salmonella* in the solution. The dispersion state of MNPs can be visually observed to qualitatively determine the presence of the target. The transverse relaxation time (*T*_2_) of the MNP_30_-S_2_ probes serves as the reporting signal for quantification, with *T*_2_ values decreasing as the concentration of *Salmonella* Typhimurium increases, achieving a detection limit as low as 1.3 × 10^2^ CFU mL^−1^. The method also shows good selectivity in the presence of non-target common foodborne pathogens such as Staphylococcus aureus and *Escherichia coli*. It not only performs well in detecting pure cultured colonies but also shows satisfactory results in detecting actual chicken samples. This strategy also has expansion potential; by designing appropriate cr-RNA and using other Cas proteins with *trans-*cleavage activities, it is expected to detect more pathogenic microorganisms. However, this strategy still relies on polymerase chain reaction (PCR) amplification, which may complicate the process, and it may require improvement in the future.

CRISPR/Cas system-based biosensors, whose output signal is magnetic transverse relaxation time, have also been explored in ultra-sensitive detection without nucleic acid pre-amplification.

Wei et al. combined the CRISPR/Cas12a system, plasmid RCA, and enzyme-catalyzed click chemistry to develop a biosensor [80]. This biosensor achieves ultra-sensitive detection of methicillin-resistant *Staphylococcus aureus* (MRSA) in food without the pre-amplification of nucleic acids. The working principle of this magnetic relaxation switching (C-MRS) biosensor is as follows: After the CRISPR system recognizes the *mecA* gene in MRSA, the *trans-*cleavage activity of Cas12a is activated, releasing ALP that is fixed on the plasmid via single-stranded DNA. The released ALP hydrolyzes the non-reducing substrate 2-phosphate-l-ascorbic acid trisodium salt (AAP) to produce ascorbic acid (AA), which triggers a click reaction between magnetic probes. The detection of MRSA is then achieved by measuring the change in the *T*_2_ of unbound magnetic probes (MNP_30_). This method achieves a detection limit as low as 16 CFU/mL, with a wide linear range (10^2^–10^6^ CFU/mL), without the need for pre-amplification and no background MRS signal. Through three levels of signal transformation and amplification, the sensitivity is significantly enhanced. The biosensor demonstrates good sensitivity and specificity in the detection of foodborne antibiotic-resistant bacteria in complex matrices, and it also has the potential to be expanded to the detection and analysis of other antibiotic-resistant bacteria. The biosensor demonstrates comparable accuracy to the traditional gold standard qPCR method, with excellent experimental stability, as evidenced by low variability in both intraday and interday measurements.

The mechanical signal is also a kind of special output signal in addition to the magnetic signal.

Zeng et al. designed a CRISPR-Cas12a-driven piezoresistive wireless biosensor for the dynamic detection of human papillomavirus (HPV)-related DNA, with convenient signal reading via smartphones [81]. Utilizing the indiscriminate cleavage ability of the CRISPR-Cas12a system on surrounding ssDNA, magnetic bead–ssDNA–gold-platinum nanoparticle conjugates (MB-ssDNA-Au@PtNPs) were introduced as signal transduction tags instead of traditional fluorescent labels to trigger a target-activated gas generation reaction. A flexible cross-shaped electrode-modified MXene-PEDOT:PSS film served as the flexible pressure sensing device, and a 3D-printed Ti_3_C_2_T_x_-PEDOT:PSS/PDMS film with randomly distributed spine-like bionic microstructures on its surface provided excellent force–electric conversion capabilities. The biosensor was connected to a Bluetooth system via integrated circuits for data transmission, processing, and storage, enabling real-time visualization of experimental results. The biosensor demonstrated good reproducibility, with intra-batch variation below 5% and inter-batch consistency across a wide concentration range of HPV-16. Compared to traditional fluorescence methods, the detection limit (15.22 pM) was lower. By converting the biomolecular recognition process into a high-pressure signal through gas generation, the pressure-sensitive device converted the pressure signal into an electrical signal, which was then transmitted to a smartphone via an integrated wireless data transmission platform. This three-step signal amplification/signal transmission strategy enhanced both portability and sensitivity.

A visual biosensor based on a CRISPR/Cas12a system-activated target-triggered SDA reaction and responsive hydrogel was designed by Feng et al. for the quantitative detection of miRNA-let-7a in human serum and cell lysates (Figure 5B) [79]. This biosensor effectively converts the concentration of miRNA-let-7a into a distance signal, with no need for complex instrumentation, allowing for quantification by simply observing distance changes with the naked eye. This feature makes it promising for rapid clinical diagnostics. The concentration of trypsin affects the hydrolysis of gelatin, which in turn determines the permeability of gelatin-treated filter paper. The differing permeability of the filter paper affects the distance the solution can travel along the cotton thread. This straightforward phenomenon allows for the cleaved ssDNA and the miRNA present in the detection system to be quantitatively detected. Within the concentration range of 10 pM to 10 nM, a logarithmic correlation is observed between the measured distance and miRNA-let-7a concentration, demonstrating excellent linearity in the detection range. This biosensor demonstrates good specificity and reproducibility.

Biosensors utilizing various CRISPR/Cas systems exhibit distinct characteristics. The CRISPR/Cas12 system, particularly Cas12a, is favored in biosensing for its *trans*-cleavage activity, high specificity, flexible PAM sequence requirements, and enhanced signal amplification. Unlike Cas9, Cas13, and Cas14, Cas12 provides greater versatility and sensitivity in DNA detection without requiring complex pre-amplification, streamlining the detection process. These attributes render it highly suitable for applications in pathogen detection, genetic mutation analysis, food safety, and related fields.

Table 1 summarizes the CRISPR/Cas system-based biosensors mentioned above.

### 3.4. Signal-Integrated CRISPR/Cas Biosensing System

#### 3.4.1. CRISPR/Cas System-Based Dual-Mode Biosensors

With the advancement of technology and the increasing demands of practical detection, people are no longer satisfied with single-signal output modes in biosensing. Consequently, many dual-mode biosensors based on CRISPR/Cas have emerged.

**Figure 6:**
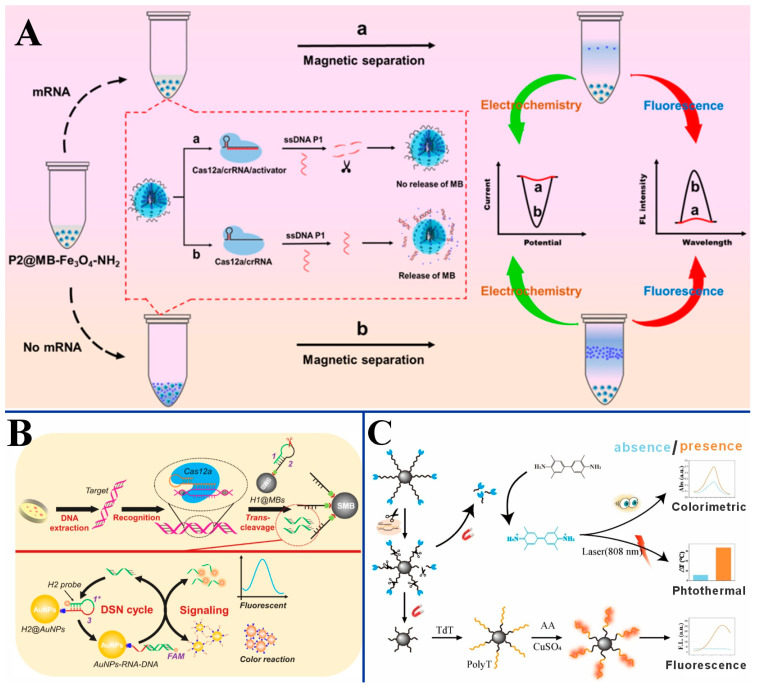
Illustration of (**A**) NEM-RCA and biosensor for *survivin* mRNA detection (a-mRNA existing; b-no mRNA existing). Reproduced with permission from [82]. Copyright 2023, American Chemical Society. (**B**) The colorimetric/fluorescent dual-mode biosensor for cfDNA detection. Reproduced with permission from [84]. Copyright 2024, Elsevier. (**C**) The CPF-CRISPR biosensor [85].

Using the CRISPR/Cas12 system, targeting survivin mRNA as the target RNA, Dong et al. developed a dual-mode electrochemical/fluorescent biosensor with Fe_3_O_4_-NH_2_ doped with MB in mesopores and surface-encapsulated with ssDNA P2 (P2@MB-Fe_3_O_4_-NH_2_) as the signal probe (Figure 6A) [82]. When the target mRNA is present, it triggers nicking endonuclease-mediated rolling circle amplification (NEM-RCA), producing a large amount of specific ssDNA that bind to the crRNA of the CRISPR system, activating the *trans-*cleavage activity of Cas12a. The free ssDNA P1 in the system is cleaved and cannot bind to the ssDNA P2 on the surface of Fe_3_O_4_-NH_2_, making it difficult to open the “biogate” to release MB. After magnetic separation, little MB remains in the supernatant, resulting in weak electrochemical and fluorescent signals. The dual-mode detection results can corroborate each other, improving the reliability and accuracy of the detection.

Deng et al. developed a dual-mode fluorescent/electrochemical biosensor for the detection of epidermal growth factor receptor (EGFR 19) [83]. This biosensor combines CRISPR/Cas12a with primer-assisted rolling circle amplification (PARCA). An activated Cas12a indistinguishably cleaves the fluorescence quencher (F-Q) probe to generate a fluorescent signal. This mode has a linear detection range from 500 fM to 10 nM. Meanwhile, the F-Q probe is replaced with a single-stranded DNA probe modified with MB at one end (SP-MB). The nanomaterial UIO-66-NH_2_ exhibits different adsorption behaviors towards intact and cleaved SP-MB, resulting in distinct electrochemical signals. The intact SP-MB produces a stronger electrochemical signal. This difference in signal allows for the detection of EGFR 19, with a detection limit of 42 aM.

Not only is the combination of fluorescence signals modes and electrochemical modes common, but colorimetric methods are also often combined with fluorescence modes.

Fareeha Arshad et al. developed a dual-mode fluorescent/colorimetric biosensor for the detection of *Salmonella* [56]. This biosensor utilizes recombinase polymerase amplification (RPA) combined with CRISPR/Cas12a technology and cerium dioxide (CeO_2_) nanozymes (NZ) that exhibit phosphatase-like and peroxidase-like activities. It can detect *Salmonella* at concentrations as low as 0.88 pg/μL (fluorescent mode) and 1.28 pg/μL (colorimetric mode)—even analyst targets in real samples of raw foods, such as eggs. The fluorescent mode employs a commonly used FAM-labeled fluorescent probe. When ssDNA is cleaved by the activated Cas12a, the fluorescent label is separated from the quencher, resulting in the generation of a fluorescent signal. Meanwhile, the colorimetric mode primarily leverages the enzyme-mimicking activities of CeO_2_. When ssDNA is cleaved by the activated Cas12a, nucleotides are released. The phosphatase-like activity of CeO_2_ NZ hydrolyzes the phosphate bonds, releasing phosphate ions (Pi). The peroxidase-like activity of CeO_2_ facilitates a colorimetric reaction through substrate oxidation, enabling visual detection. The intensity of the observed color is proportional to the concentration of the target DNA molecules.

Zhao et al. designed a colorimetric/fluorescent dual-mode biosensor for detecting cell-free DNA (cfDNA), which leverages the specific recognition capabilities of the CRISPR-Cas12a system, the selective cleavage of DNA/RNA heteroduplexes by double-stranded nuclease (DSN), and two hairpin probes modified with both DNA and RNA fragments on magnetic beads (H1@MBs) and gold nanoparticles (H2@AuNPs), respectively (Figure 6B) [84]. The detection process does not require pre-amplification steps. When the target DNA sequence is present, the crRNA recognizes it and activates the *trans-*cleavage activity of Cas12a, which cleaves the H1@MBs probe. The released RNA fragments then hybridize with the RNA part of H2@AuNPs, which are labeled with a fluorophore and a quencher, forming a DNA/RNA heteroduplex. This heteroduplex is subsequently hydrolyzed by DSN, freeing the fluorophore and restoring the fluorescent signal. The cleaved AuNPs, now only modified with short DNA strands, can be used for the colorimetric reaction. As the concentration of the target gene increases, the color of the solution changes from red to blue.

Xu et al. designed an electrochemiluminescence (ECL)/electrochemical (EC) dual-mode biosensor, which cleverly utilizes the principle of “competitive binding” and the electrostatic differences in interactions between probes with different charges and DNA to detect ochratoxin A (OTA) in traditional Chinese medicine [86]. The ssDNA used in this biosensor is unique, as it is not only complementary to the crRNA in the CRISPR-Cas12a system but also serves as an aptamer for OTA. Therefore, when OTA is present in the sample, the ssDNA preferentially binds to OTA, inhibiting the activity of CRISPR-Cas12a. As a result, the hairpin DNA immobilized on the gold electrode is not cleaved, and its negative charge attracts Ru(bpy)_3_^2+^ (ECL probe) while repelling [Fe(CN)_6_]^3−/4−^ (EC probe). This leads to an enhanced ECL signal and a weakened current signal, with detection limits of 0.29 pg/mL (ECL) and 0.37 pg/mL (EC), respectively.

These biosensors integrate two distinct signal readout modes to provide complementary detection results, enhancing reliability and offering user-friendly features. However, cross-talk between the two signal modes may occur, potentially affecting detection accuracy. Additionally, the lack of standardized protocols for dual-mode biosensors hinders their widespread adoption and reproducibility. Compared to single-signal biosensors, dual-mode biosensors provide cross-verified results, reducing the risk of false readings, and their combination of two signal modes enables detection in complex matrices, like blood. Therefore, future research should focus on developing standardized protocols for dual-mode biosensor fabrication and operation to improve reproducibility and comparability. Additionally, integrating dual-mode biosensors with microfluidic platforms can enable high-throughput, automated analysis, making them highly suitable for POCT settings.

#### 3.4.2. CRISPR/Cas System-Based Multimodal Biosensors

In addition to dual-mode CRISPR/Cas system-based biosensors with two signal output modes, researchers are also continuously exploring the integration of more signal output methods into a single biosensor.

Wu et al. proposed a multimodal biosensor that combines CRISPR/Cas12a technology with the enzyme-like activity of G-quadruplex (G4) to achieve colorimetric/SERS/fluorescent detection of AFB1 in food, with detection limits of 0.85, 0.79, and 1.65 pg⋅mL^−1^, respectively [87]. The designed special cDNA sequence is not only complementary to crRNA but also binds with the aptamer of AFB1. Therefore, in the presence of the target, the cDNA cannot bind to the aptamer of AFB1. After pairing with crRNA, the *trans-*cleavage activity of Cas12a is activated, disrupting the G4-DNAzyme (the G4/hemin complex exhibits peroxidase-like activity, catalyzing the oxidation of TMB to produce corresponding SERS and fluorescence signals and causing a noticeable color change from blue to yellow under acidic conditions). The fluorescence intensity of blue TMBox, the color intensity of yellow TMBox, and the SERS intensity under the AgNPs substrate in the solution are all weak.

CRISPR/Cas system-based biosensors that rely solely on photothermal signal detection are scarce, but this can be used as a signal output mode in multimodal biosensors.

Zheng et al. developed a “CPF-CRISPR” colorimetric/photothermal/fluorescent multimodal biosensor for the detection of drug-resistant bacteria, such as methicillin-resistant *Staphylococcus aureus* (MRSA), with a detection limit of 10^1^ CFU/mL (Figure 6C) [85]. This biosensor leverages the *trans-*cleavage activity of CRISPR/Cas12a, the enzyme-catalyzed reaction of TMB, and the fluorescence properties of copper nanoclusters formed on a DNA scaffold. A signal probe, MNPs-ssDNA-HRP, is designed as the substrate. In the presence of the target bacterial DNA, CRISPR/Cas12a is activated, cleaving the signal probe and releasing HRP. The free HRP can catalyze the oxidation of TMB, resulting in a visible color change from colorless to yellow. Under 808 nm near-infrared (NIR) laser irradiation, the solution turns blue and emits a photothermal signal. The isothermal amplification reaction mediated by TdT (terminal deoxynucleotidyl transferase, which catalyzes the synthesis of poly-T by adding dTTPs to the 3′ hydroxyl end of the primer) is initiated by short DNA strands remaining on magnetic beads. In the presence of AA, Cu^2+^ forms CuNCs, using poly-T as a scaffold and generating a fluorescent signal, with the signal intensity correlating with the length of the poly-T.

Although signal-integrated CRISPR/Cas biosensing systems can output multiple signals, there are large differences in signal sensitivity, stability, and other performance indicators under various modes. In addition, the signal combination modes of dual-modal and multi-modal imaging are relatively fixed, and it is still difficult to combine any two or more signal output modes.

### 3.5. Structure-Integrated CRISPR/Cas Biosensing System

#### 3.5.1. CRISPR/Cas Biosensors Integrated with Lateral Flow Technology

Lateral flow biosensor utilizes the movement of the sample along the test strip, with different capture substances set in the test and control line areas to achieve detection. It is low cost, easy to operate, and highly compatible, making it one of the most successful commercial detection tools. Consequently, many CRISPR/Cas system-based biosensors are also integrated with lateral flow technology.

**Figure 7:**
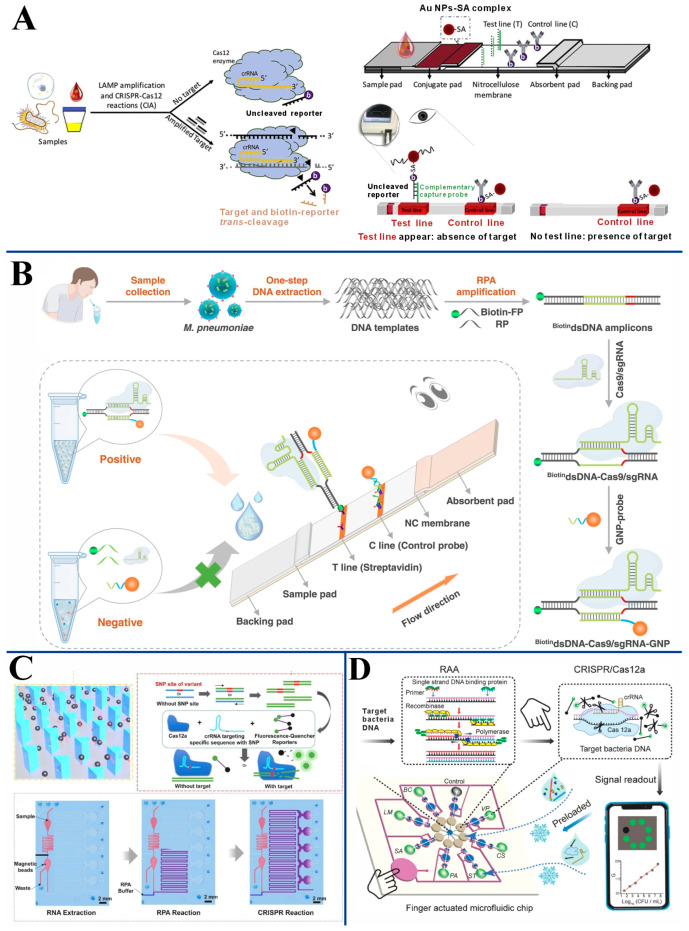
The working mechanism of (**A**) CIA-based LFB. Reproduced with permission from [88]. Copyright 2020, Elsevier. (**B**) Established approach for *Mycoplasma pneumoniae* detection. Reproduced with permission from [91]. Copyright 2023, Elsevier. (**C**) The designed LOC-CRISPR system. Reproduced with permission from [92]. Copyright 2023, Elsevier. (**D**) The microfluidic biosensor for multiplexed foodborne pathogen detection. Reproduced with permission from [93]. Copyright 2023, Elsevier.

Omar Mukama et al. designed a lateral flow biosensor (LFB) that utilizes the CRISPR/Cas system and loop-mediated isothermal amplification (CIA) technology to detect Pseudomonas aeruginosa, capable of detection even at single-copy clone concentrations in samples. Streptavidin-modified gold nanoparticles (AuNP-SA) are used as reporter molecules (Figure 7A) [88]. These molecules react with biotin-modified ssDNA reporter genes to form AuNP-SA-biotin-ssDNA complexes. These complexes are captured by probes on the test line (T line), while excess AuNP-SA reacts with biotin-modified antibodies on the control line (C line) to verify the performance of the biosensor. When the acyltransferase gene of Pseudomonas aeruginosa is present, the Cas effector protein is activated and cleaves the target and any surrounding ssDNA reporter genes. The DNA probe complementary to the reporter sequence fixed at the T line cannot produce a corresponding signal, but there is still a signal at the C line. Visual inspection of the absence or presence of the T line and C line can determine whether Pseudomonas aeruginosa infection is present. By changing the corresponding DNA sequences, they also used a similar sensing mechanism to detect human papillomavirus HPV16 and HPV18 [89]. Similarly, when the target gene is present, there is a signal at the C line but not at the T line; when the target gene is absent, both the C line and T line appear. Even mismatches in individual bases of the target gene do not affect detection.

Most CRISPR/Cas system-based biosensors combined with lateral flow technology need to be used in combination with nucleic acid pre-amplification technology. Except for loop-mediated isothermal amplification, RPA is also commonly used.

Wang et al. designed a lateral flow biosensor for analyzing DNA methylation in human serum, which combined a dual methylation-sensitive endonucleases system (*BstUI*/*HhaI*) with the CRISPR/Cas13a system and RPA for the first time [90]. This innovative enzyme-cutting strategy, named DESCS, can effectively distinguish methylated DNA from an excess of unmethylated DNA, even at methylation levels as low as 0.01%, and can visually identify methylated DNA at concentrations as low as 200 aM. Taking the SEPT9 gene (SEPT9-mC), which is highly methylated in human colorectal cancer (CRC), as an example, it cannot be recognized by BstUI/HhaI enzymes compared to unmethylated SEPT9-C, thus remaining intact. The intact SEPT9-mC target triggers the RPA reaction, producing a large amount of the dsDNA product bound to the T7 promoter, which is then transcribed into ssRNA by T7, activating the ssRNase activity of Cas13a. This results in the cleavage of the lateral flow reporter gene (a short ssRNA labeled with 5′-FAM and 3′-biotin). The uncleaved reporter gene is specifically captured at the C line by binding with Au NP-anti-FAM-antibody and streptavidin. Meanwhile, the cleaved short RNA fragments labeled with FAM form complexes with the Au NP-anti-FAM-antibody, which are captured by antibodies at the T line, producing a dark line. The higher the concentration of methylated DNA, the darker the T line. Unmethylated DNA, on the other hand, only produces a single line at the C line.

The lateral flow biosensor designed by Zhu et al. utilized the CRISPR/Cas9 system without *trans-*cleavage activity to detect Mycoplasma pneumoniae. When the target gene is present, the RPA reaction incorporates a biotin label into the target gene, resulting in a large amount of dsDNA amplicons (Biotin-dsDNA) (Figure 7B) [91]. These amplicons are specifically recognized by the Cas9 protein, exposing sequences that can complementarily pair with the GNP probe, forming a biotindsDNA-Cas9/sgRNA-GNP complex. As this complex passes through the T line, it is captured by pre-fixed streptavidin, displaying a red color. On the C line, the probe captures free GNPs, showing color regardless of the presence of the target gene. This biosensor can complete the detection process within 30 min, and the T line signal exhibits a good linear relationship with the copy number of targets over a wide range, from 3 × 10^2^ to 3 × 10^6^.

CRISPR/Cas biosensors integrated with lateral flow technology are cost-effective, user-friendly, and portable, making them ideal for POCT. The integration of CRISPR/Cas systems significantly enhances their specificity and sensitivity, enabling the detection of targets such as pathogens and methylated DNA through simple modifications of guide RNA (crRNA) and probe sequences. LFBs provide rapid, visual results interpretable without specialized equipment, enhancing accessibility in resource-limited settings. However, many CRISPR/Cas-based LFBs require nucleic acid amplification for high sensitivity, complicating workflows, and visual readouts are often qualitative or semi-quantitative. Future advancements should focus on multiplexed detection, smartphone-based quantification, and amplification-free designs to improve versatility and streamline performance.

#### 3.5.2. CRISPR/Cas Biosensors Integrated with Microfluidic Technology

Microfluidic technology offers significant advantages over traditional laboratory tests due to its requirement of small sample volumes, high fluidity, and capability for multiplexed control [94]. To achieve high-sensitivity detection of multiple low-concentration targets simultaneously, researchers have integrated microfluidic technology with CRISPR/Cas biosensors.

Shen et al. developed a microfluidic biosensor named LOC-CRISPR, which can identify multiple common respiratory viruses (such as SARS-CoV-2, H1N1, etc.) and their variants simultaneously and specifically (Figure 7C) [92]. RNA from the sample is extracted using magnetic beads that can adsorb RNA and a serpentine channel microarray. After undergoing an RPA reaction, CRISPR-specific recognition is performed in five parallel reaction chambers. Special structures and materials are used to prevent cross-interference. In the presence of the target DNA, the nuclease and *trans-*cleavage activities of Cas12a are activated, leading to the cleavage of the ssDNA reporter probe. The fluorescent molecule FAM and the quencher BHQ attached to both ends of the probe are separated, producing a fluorescent signal at 520 nm when irradiated with a 480 nm laser. The multi-chamber design also provides information about the variant type of the virus present in the sample.

Xing et al. designed a microfluidic biosensor that combines the CRISPR/Cas12a system with technology, enabling the simultaneous detection of multiple common foodborne pathogens with LOD below 500 CFU/mL (Figure 7D) [93]. The collected fluorescent signals can be gathered and processed by a smartphone, making the process more convenient and efficient. The sensing principle still employs the commonly used fluorescence recovery mechanism (presence of target molecule—activation of Cas12a—cleavage of ssDNA probe—fluorescence recovery). A unique feature of this design is the use of a finger-actuated, one-way control valve that controls the unidirectional flow of RAA products, preventing potential cross-contamination between the RAA and CRISPR recognition processes.

Like the above biosensors based on fluorescence output signals, which are based on electrochemical signal output modes, these also use microfluidic structures to prevent possible interference.

Zhang et al. designed a CRISPR/Cas13a-mediated microfluidic electrochemical biosensor that can detect the SARS-CoV-2 virus within 25 min, with an LOD of 10 aM [95]. To prevent interference from nucleases that may degrade crRNA in the sample, the microfluidic module separates the virus lysis and nuclease inactivation steps from the subsequent CRISPR/Cas13a recognition and detection steps. The screen-printed gold electrode is functionalized with MB reporter molecules connected by short ssRNA chains composed solely of uracil (poly20). In the presence of SARS-CoV-2 RNA, the *trans-*cleavage activity of Cas13a is activated, leading to the cleavage of poly20. This reduces the MB labeling, resulting in a decrease in the electron transfer rate. Consequently, the square wave voltammetry (SWV) detects a reduced redox peak current.

CRISPR/Cas biosensors integrated with microfluidic technology enable high-throughput, specific detection of targets using minimal sample volumes. The modular design of microfluidic platforms facilitates efficient workflows and prevents cross-contamination, while diverse signal output modes enhance versatility. These biosensors deliver rapid results within minutes, making them ideal for POCT in resource-limited settings, with reduced reagent costs and waste. However, the fabrication of microfluidic devices with integrated CRISPR/Cas systems is technically challenging and costly, and some designs rely on external equipment for signal readout, potentially limiting accessibility. Future advancements should focus on simplifying fabrication and integrating portable readout systems to broaden their application.

#### 3.5.3. CRISPR/Cas System-Based Portable Biosensors Integrated with Glucometer

People are pursuing more portable and real-time biological detection methods. Developing new, simpler biosensing mechanisms is one approach, but utilizing existing mature devices to simplify the detection process is equally effective. As one of the most common commercial biosensors, the glucometer is naturally the first choice for researchers.

**Figure 8:**
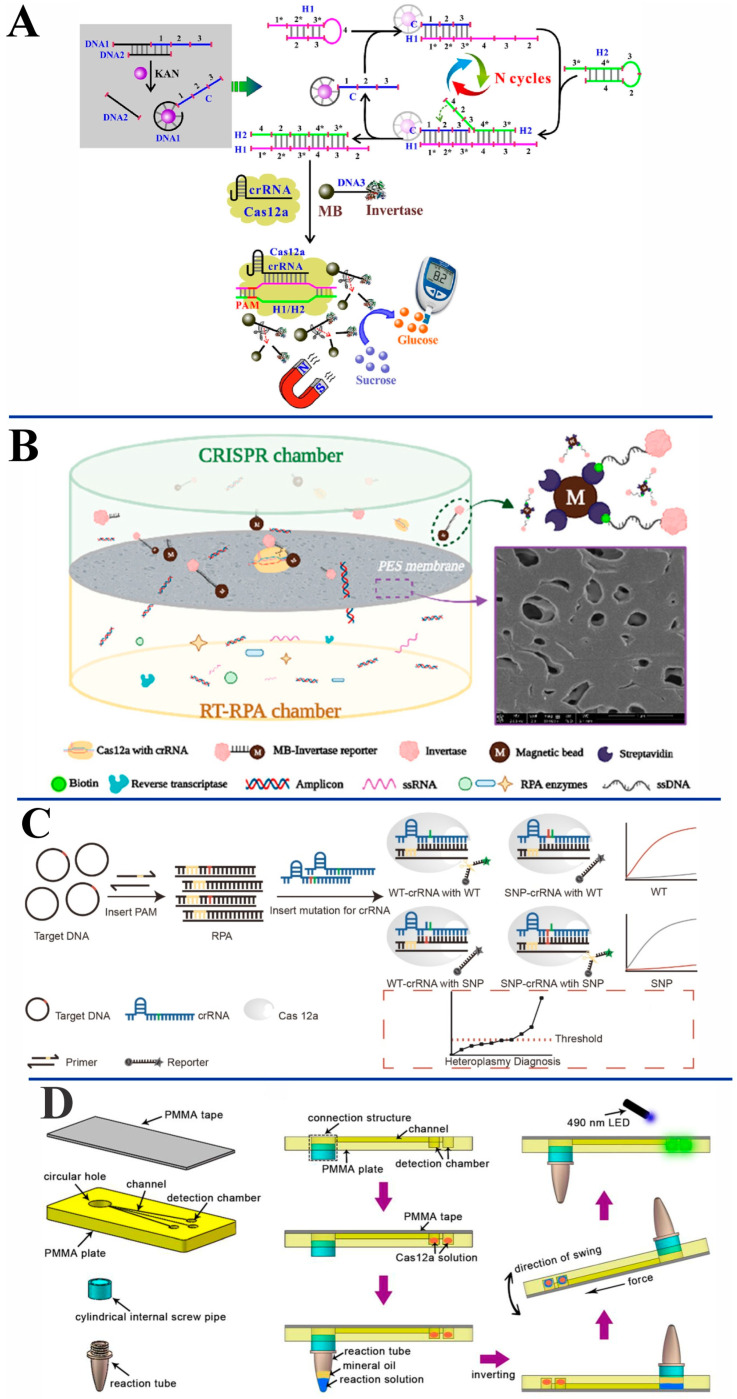
Principle of (**A**) a portable biosensor for on-site kanamycin detection. Reproduced with permission from [96]. Copyright 2023, Elsevier. (**B**) CRISPR-MCR biosensor. Reproduced with permission from [97]. Copyright 2023, American Chemical Society. (**C**) mtDNA disease diagnosis based on RatioCRISPR. Reproduced with permission from [99]. Copyright 2023, Elsevier. (**D**) CRISPR/Cas12a-based portable biosensor. Reproduced with permission from [100]. Copyright 2020, Elsevier.

Chen et al. developed a portable kanamycin (KAN) biosensor that utilizes an existing glucometer as the detection device and incorporates the CRISPR-Cas12a system to amplify the signal (Figure 8A) [96]. In the presence of KAN, it interacts with specific single-stranded DNA fragments, releasing complementary sequences that can trigger the recognition and hybridization of hairpin probes H1 and H2. This process generates DNA fragments that can be recognized by the crRNA of the CRISPR/Cas system, activating the *trans-*cleavage activity of Cas12a. This activity cleaves the single-stranded DNA connecting magnetic beads and invertase. After magnetic separation, the invertase remains in the solution and can convert sucrose into glucose, which is ultimately detected by the glucometer. This biosensor has a broad detection range (1 pM~100 nM), can detect complex samples, and has strong anti-interference capabilities against non-target antibiotics.

Inspired by the enzyme cascade reactions in living cells, Li et al. designed a CRISPR-mediated cascade reaction (CRISPR-MCR) biosensor (Figure 8B) [97]. This biosensor uses a nanoporous membrane to separate the RPA reaction of the target nucleic acid sequence from the recognition and cleavage reaction of the CRISPR/Cas system. The small-pore-sized porous PES membrane is highly hydrophilic, is chemically resistant, and has low protein-binding properties, confining different functional enzymes to their respective reaction chambers effectively. This prevents signal interference from the RPA reaction solution or other biomolecules, reducing the occurrence of false positives and enabling efficient CRISPR-MCR detection. When HIV nucleic acids are present and amplified in the corresponding chamber, the concentration difference allows the amplicons to pass through the nanopores into the CRISPR/Cas system reaction chamber. The activated Cas12a then cleaves the MB-ssDNA-invertase. Magnetic beads are retained in the reaction chamber by a neodymium disk magnet located below the biosensor. Bubbles and water transfer the free invertase to the catalytic chamber, where it hydrolyzes sucrose to produce glucose, which is ultimately detected digitally using a personal glucometer. In practical applications, the CRISPR-MCR biosensor can detect 43 copies of HIV DNA plasmids and 200 copies of HIV RNA, demonstrating clinical performance comparable to RT-PCR methods.

Not only can positively correlated glucose molecular signals be exploited but also negatively correlated ones.

Zhou et al. developed a simple biosensor targeting *Salmonella* using the CRISPR/Cas12a system and an existing glucometer [98]. The invA gene serves as the target gene, and through steps including recombinase-aided amplification (RAA), Cas12a *trans-*cleavage of ssDNA, and sucrose hydrolysis, the presence of *Salmonella* is converted into a glucose signal that can be read by a personal glucometer. This allows for rapid detection with sensitivity as low as 5 CFU per reaction. Unlike most sensors where the glucose concentration detected by the glucometer increases with the initial nucleic acid concentration, this biosensor exhibits a negative correlation between glucose concentration and the initial nucleic acid concentration. This is because the researchers did not use the method of linking magnetic beads with glucose invertase via ssDNA. Instead, magnetic beads and invertase are separately modified on complementary ssDNA strands. Initially, only magnetic beads with ssDNA are added to the system. If the target gene is present, the activated Cas12a will cleave the ssDNA indiscriminately, preventing the magnetic beads from connecting with the invertase and participating in the subsequent sucrose hydrolysis reaction, resulting in a lower signal. This approach avoids potential spatial effects that could arise from directly linking MBs and invertase via ssDNA during Cas12a cleavage. In the mixed detection of standard *Salmonella*, clinically isolated *Salmonella*, and non-*Salmonella* samples, this biosensor demonstrates good specificity, with only samples containing *Salmonella* showing a decrease in glucose signal. The signal difference between samples containing *Salmonella* and those without is significant. The biosensor also shows good spiked recovery rates in various food samples susceptible to *Salmonella* infection.

These biosensors leverage commercially available glucometers, offering the distinct advantages of portability and real-time detection. Glucose signals can be designed to exhibit either positive or negative correlations with target concentration, providing flexibility in biosensor design. The use of widely accessible glucometers significantly reduces system costs, making these biosensors economically viable for widespread use. However, current designs are primarily limited to single-target detection, although ongoing advancements aim to enable multiplexed analysis. Future developments should focus on expanding multiplexing capabilities while maintaining the simplicity and cost-effectiveness that make these biosensors highly practical for point-of-care applications.

#### 3.5.4. Other CRISPR/Cas System-Based Chip-Type Biosensors

With the increasing demand for detection, integration may become one of the main research directions in the future. Consequently, many researchers have also explored CRISPR/Cas system-based chip-type biosensors.

Devora Najjar et al. designed a multiplexed electrochemical biosensor chip that can simultaneously detect SARS-CoV-2 RNA and anti-SARS-CoV-2 immunoglobulins in saliva [101]. A portion of the collected saliva sample undergoes isothermal nucleic acid amplification and CRISPR/Cas12 system recognition and *trans-*cleavage, followed by RNA detection using an enzyme-linked immunosorbent assay (ELISA). The signal obtained from the electrochemical platform is inversely proportional to the concentration of the target RNA. Meanwhile, another untreated portion is delivered by a peristaltic pump to other reaction chambers, where an affinity-based antigen sandwich ELISA is performed to detect the corresponding antibodies. Different subtypes of SARS-CoV-2 antibodies bind to the antigens and secondary antibodies fixed on the chip surface in different pathways, leading to various increases in electrochemical signals. Finally, clinical samples are tested for four categories: (1) RNA-negative, antibody-negative; (2) RNA-positive, antibody-positive; (3) RNA-negative, antibody-positive; and (4) RNA-positive, antibody-negative. All results show high discrimination (*p* < 0.0001), with ultra-low background signals between positive and negative samples.

Effective biosensing detection can be achieved by using ratiometric signals instead of absolute quantification. Wu et al. designed a ratio-type biosensor chip named “RatioCRISPR” to detect the heterogeneity levels of single-nucleotide polymorphisms (SNPs) in mitochondrial DNA (mtDNA) using the CRISPR/Cas12a system (Figure 8C) [99]. The sensitivity and specificity of this biosensor are enhanced through the strategic introduction of mismatched nucleotides near critical recognition sites. The ratio measurement system consists of two different crRNAs: one is SNP-crRNA, which is complementary to the mutant type and mismatched with the wild type, and the other is WT-crRNA, which is the opposite (non-complementary to the mutant type and matched with the wild type). Both crRNAs detect the same target simultaneously. When the mutant target is present, the SNP-crRNA generates a high signal, and the WT-crRNA generates a low signal; conversely, when encountering the wild-type target, the signals are reversed. This differential signaling mechanism enables the rapid assessment of target heterogeneity levels. This design not only improves the sensitivity of detection but also enhances the ability to distinguish signals from different genotypes, making the detection of SNPs in mitochondrial DNA more accurate and efficient.

The exceptional electrical and surface properties of graphite also make it advantageous as CRISPR/Cas system-based chip-type biosensors.

Reza Hajian et al. reported a FET biosensor chip based on functionalized graphene, called CRISPR-Chip, which uses CRISPR technology to detect target sequences in intact genomic samples [102]. The chip comprises a complex of catalytically deactivated Cas9 protein (dCas9) and single-guide RNA (sg-RNA) designed for specific sequences. This complex is anchored to the surface of the graphene channel using molecular linkers, forming a dRNP complex that serves as the channel between the source and drain electrodes of the transistor. The high specificity and sensitivity of CRISPR-Cas allow it to scan intact genomic samples and hybridize with specific complementary sequences. The high carrier mobility of graphene makes it extremely sensitive to the adsorption and interaction of surface-charged molecules. Therefore, when the target sequence is present, the dRNP complex can quickly locate and bind to it, altering the electrical properties of the gFET and generating an electrical signal output. The percentage change in the current (I-response) within 15 min is significantly higher than in samples lacking the target sequence. For clinical DNA samples with common gene mutations in patients with Duchenne muscular dystrophy (DMD), the detection limit is 1.7 fM, which is lower than previously reported non-amplification technologies. In addition to detecting other target nucleic acids by changing the type of Cas protein, CRISPR-Chip also has the capability to provide information on the affinity between sgRNA and various Cas proteins. This chip is helpful for predicting and validating the formation efficiency of CRISPR complexes under various conditions as well.

Zheng et al. developed a biosensor chip named CRISPR-SPR-Chip by combining the specific recognition ability of the CRISPR/Cas system, surface plasmon resonance (SPR) technology, and a 2D graphdiyne structure [103]. This chip can be used for the clinical screening of patients with certain hereditary diseases caused by gene mutations, such as DMD. The introduction of graphdiyne films not only provides the chip with a better protein (i.e., dCas9 nuclease) loading capacity but also enhances the SPR signal response due to its electromagnetic field coupling effect with the Au metal layer. The biosensor exhibits high sensitivity, with a detection limit of 1.3 fM for recombinant plasmids with only three nucleobase mutations. It also offers rapid detection without the need for pre-amplification steps, producing significant positive results from patient samples within 5 min.

Wu et al. developed a chip-based visual biosensor for the convenient detection of genetically modified (GM) soybean flour, capable of simultaneously detecting the CaMV35S promoter and the lectin gene (Figure 8D) [100]. This biosensor combines the CRISPR/Cas12a system with loop-mediated isothermal amplification (LAMP) on a polymethyl methacrylate (PMMA) chip. When the target gene is detected, the ssDNA nuclease activity of the Cas12a is activated, cleaving the surrounding ssDNA fluorescent probes. Upon illumination with a 490 nm LED light, a visual detection result is obtained; green fluorescence indicates a positive sample, while black fluorescence indicates a negative sample. Since the entire chip-based biosensor operates in a closed system, it effectively addresses the issues of amplicon contamination and non-specific amplification that previous methods might encounter. This biosensor chip can detect GM content as low as 0.1% in soybean flour and can also differentiate whether food samples contain only one of the two transgenic components, the CaMV35S promoter or the lectin gene, demonstrating good sensitivity and specificity.

Chip-type biosensors integrate CRISPR/Cas systems with multiple functional components into a compact platform, enabling high-throughput and multiplexed detection. Their miniaturized design enhances automation and portability, making them ideal for POCT. However, the complexity of their structural design poses challenges for fabrication and scalability. These biosensors are particularly well suited for integration with smartphone-based readouts, offering user-friendly, real-time detection capabilities.

Structure-integrated CRISPR/Cas biosensing systems are generally simpler in terms of readout and on-site detection operations, but they still mostly require nucleic acid pre-amplification and are complex in their structure design and combination.

## 4. Conclusions and Prospects

Although various types of CRISPR/Cas biosensors have been studied and are continuously being developed, most constructed biosensors are still combined with methods such as fluorescence, colorimetry, and electrochemistry. There is still insufficient exploration of their combinations with many novel biosensing methods. The use of the CRISPR/Cas system is concentrated on the *trans-*cleavage activity of a few Cas proteins, and whether more effects can be utilized is worth exploring.

Research on the intelligence, simplicity, and wearable ability of biosensors is gradually becoming a focal point. However, there is still little exploration of CRISPR/Cas system-based biosensors in these areas, which may be a direction worth pursuing in the future. Due to the limitation of target molecule concentration, many CRISPR/Cas system-based biosensors still rely on various nucleic acid amplification methods, increasing the complexity of the detection process. Although many non-amplification CRISPR/Cas system-based biosensors have emerged, the question of how to better design sensing mechanisms and simplify the detection process is still worth considering. Among the signal readout modes, electrochemical and fluorescence readouts are currently the most promising for non-amplification CRISPR/Cas system-based biosensors due to their high sensitivity, portability, and compatibility with point-of-care applications. However, SERS- and FET-based readouts also hold significant potential for ultra-sensitive and multiplexed detection. Enhancing the multiplexing capabilities of CRISPR/Cas system-based biosensors and developing all-in-one biosensors are essential for advancing their practical applications.

Each CRISPR/Cas biosensor based on optical, electrical, magnetic, and mechanical output signals has their own characteristics, but no CRISPR/Cas biosensors based on acoustic output signals have yet been found. It is hoped that in the future, more convenient and intelligent CRISPR/Cas system-based biosensors will provide more convenience to life.

## Figures and Tables

**Table 1:** Recent studies of CRISPR/Cas system-based biosensors.

Readout	CRISPR	Preamplification	Target	LOD	Ref.
Electrochemical	Cas12a	Without	HIV-16	100 fM	[39]
	Cas13a	Without	miRNA-19b	10 pM	[40]
	Cas13a	Not mentioned	circRNA	0.089 fM	[41]
	Cas12a	Not mentioned	PD-L1^+^ exosomes	38 particles/μL	[46]
	Cas12a	Target-triggered EXPAR-mediated transcription amplification reaction	miRNA-155	31.6 aM	[47]
Field-Effect Transistor	Cas12a	Without	HIV-16 *Escherichia coli*	1 aM 10 aM	[49]
	Cas13a	Without	SARS-CoV-2 variants	1 cp/μL	[42]
	Cas13a	Without	CoV, HCV	1.56 aM	[50]
Photoelectrochemical	Cas12a	Not mentioned	CEA Kana	0.045 fg/mL 0.34 pM	[43]
	Cas12a	Not mentioned	HPV-16	1.0 pM	[53]
	Cas12a	Not mentioned	miRNA	36 aM.	[54]
Fluorescent	Cas12a	Not mentioned	DDVD	0.135 ng mL^−1^	[62]
	Cas14a	Not mentioned	Cd^2+^ Histamine AFB1 Thrombin ATP	4 nM 30 nM 16 nM 36 nM 80 nM	[63]
	Cas12a	RCA and HCR	miR-155 miR-10b miR-21	33 fM 41 fM 79 fM	[64]
Colorimetric	Cas14a	Competitive dissociation and EXPAR	CK- MB	0.355 pM	[65]
	Cas12a	Not mentioned	miR-205 and miR-944	36.4 fM	[66]
	Cas12a	Not mentioned	HBV	0.5 pM	[68]
Chemiluminescence	Cas12a	HCR amplifying	HPV-16	3 pM	[67]
	Cas13a	RT-RAA	H7N9	19.7 fM	[70]
	Cas12a	RCA reaction	miRNA-21	16 aM	[69]
Electrochemiluminescence	Cas12a	Not mentioned	Dam MTase	23.4 mU/mL	[71]
	Cas12a	SDA and dual-particle three-dimensional DNA rollers	Ec-16S rDNA	27.29 aM	[72]
	Cas13a	Entropy-driven triggered T7 amplification	SARS-CoV-2	7.39 aM	[73]
	Cas12a	PCR	pufferfish COI gene	0.1%	[76]
Surface-Enhanced Raman Scattering	Cas12a	Without	HBV HPV-16 HPV-18	1 aM	[74]
	Cas12a	LAMP	DNA	224 aM	[77]
	Cas12a	Without	SARS-CoV-2	1 aM	[75]
Magnetic Relaxation Switching	Cas12a	PCR	Salmonella	1.3 × 10^2^ CFU mL^−1^	[78]
	Cas12a	Without	MRSA	16 CFU/mL	[80]
Visual Signal	Cas12a	Not mentioned	HPV-16	15.22 pM	[81]
	Cas12a	Target-triggered SDA reaction	miRNA-let-7a	6.28 pM	[79]

## Data Availability

Not applicable.

## List of Abbreviations

Abbreviations	Meaning
acDNA	activator DNA
AChE	acetylcholinesterase
AFB1	aflatoxin B1
ALP	alkaline phosphatase
ALP-SA	streptavidin-labeled alkaline phosphatase
AMI	acute myocardial infarction
Aptavator	aptamer-activator
ATCh	acetylcholine
AuNFs	gold nanoflowers
AuNPs	gold nanoparticles
Cas	CRISPR-associated proteins
CAVRED	CRISPR-based Amplification-free Viral RNA Electrical Detection
CeO_2_	cerium dioxide
cfDNA	cell-free DNA
CFU	colony-forming units
CK-MB	creatine kinase-MB
CL	chemiluminescence
CNP	charge neutral point
COI	cytochrome oxidase subunit I
CoV	coronavirus
CRC	colorectal cancer
CRISPR-MCR	CRISPR-mediated cascade reaction
crRNA	CRISPR RNA
cTn I	cardiac troponin I
CVDs	cardiovascular diseases
DA	dopamine
Dam MTase	DNA adenine methylation methyltransferase
dCas9	deactivated Cas9 protein
DMD	Duchenne muscular dystrophy
Ec-16S rDNA	Escherichia coli 16S rDNA
ECL	electrochemiluminescence
ELISA	enzyme-linked immunosorbent assay
EXPAR	exponential amplification
Fc	ferrocene
FET	field-effect transistor
FNAs	framework nucleic acids
GCE	glassy carbon electrode
G-FET/gFET	graphene-based FET
GM	genetically modified
GO	graphene oxide
GOx	glucose oxidase
G4	G-quadruplex
HBV	hepatitis B virus
HCR	hybridization chain reaction
HCV	hepatitis C virus
HPV	human papillomavirus
intDNA	initial DNA
ITO	indium tin oxide
Kana/KAN	kanamycin
LAMP	loop-mediated isothermal amplification
LFB	lateral flow biosensor
LOD	limit of detection
MB	methylene blue
miRNA	microRNA
miRNA-21	microRNA-21
MOF	metal-organic framework
MRSA	methicillin-resistant Staphylococcus aureus
mtDNA	mitochondrial DNA
NIR	near-infrared
NPs	nanoparticles
NZ	nanozymes
OPs	organophosphorus pesticides
OTA	ochratoxin A
PAM	Protospacer Adjacent Motif
PARCA	primer-assisted rolling circle amplification
PB	Prussian Blue
PCR	polymerase chain reaction
PD	polymer dot
PD-L1^+^	programmed death-ligand 1 protein positive
PEC	photoelectrochemical
PFS	protospacer flanking site
Pi	phosphate ions
PMMA	polymethyl methacrylate
POCT	point-of-care testing
QDs	quantum dots
RAA	recombinase-aided amplification
RCA	rolling circle amplification
RGO	reduced graphene oxide
RPA	recombinase polymerase amplification
RT-RAA	reverse transcription recombinase-aided amplification
SA-HRP	streptavidin–horseradish peroxidase
SDA	strand displacement amplification
SERS	surface-enhanced Raman scattering/surface-enhanced Raman spectroscopy
SNPs	single nucleotide polymorphisms
SPR	surface plasmon resonance
ssDNA	single-stranded DNA
SWV	square wave voltammetry
TCh	thiocholine
TMSDR/TSDR	toehold-mediated strand displacement reaction
2D COF NSs	2D layered structure of covalent organic framework nanosheets
